# Supplementary material for: Diverse patterns of correspondence between protist metabarcodes and protist metagenome-assembled genomes
Source: PLoS One. 2024 Jun 6;19(6):e0303697. doi: 10.1371/journal.pone.0303697 (PMC11156365; doi:10.1371/journal.pone.0303697)
Supplement: S2 File — (ZIP) [file pone.0303697.s002.zip › S1_table.pdf]

Taxonomic subset name	SMAG taxonomy "genre"	Count	V9 "supergroup"	Count	V9 "taxogroup_1"	Count	V9 "taxogroup_2"	Count	V9 "taxogroup_3"	Count	V9 "genus"	Count
Attheya	Attheya	1	Stramenopiles	10648	Ochrophyta	6926	Diatomeae	5430	Biddulphiophyceae	128	Attheya	60
Aureococcus	Aureococcus	6	Stramenopiles	10648	Ochrophyta	6926	Pelagophyceae	119	Pelagomonadales	84	Aureococcus	11
Bathycoccus	Bathycoccus	8	Chloroplastida	7436	Chlorophyta	7089	Mamiellophyceae	633	Bathycoccaceae	19	Bathycoccus	5
Cafeteria	Cafeteria	1	Stramenopiles	10648	Opalozoa	1228	Bicosoecida	230	core-bicosoecids	205	Cafeteria	26
Chaetoceros	Chaetoceros	11	Stramenopiles	10648	Ochrophyta	6926	Diatomeae	5430	Mediophyceae	2325	Chaetoceros	632
Chloroparvula	chloroparvula	7	Chloroplastida	7436	Chlorophyta	7089	Chloropicophyceae	138	Chloropicophyceae	138	Chloroparvula	58
Chloropicon	Chloropicon	11	Chloroplastida	7436	Chlorophyta	7089	Chloropicophyceae	138	Chloropicophyceae	138	Chloropicon	80
Cylindrotheca	Cylindrotheca	2	Stramenopiles	10648	Ochrophyta	6926	Diatomeae	5430	Bacillariophyceae	1929	Cylindrotheca	11
Emiliania	Emiliania	5	Haptophyta	953	Prymnesiophyceae	746	Prymnesiophyceae	746	Isochrysidales	7	Emiliania	0
Florenciella	Florenciella	3	Stramenopiles	10648	Ochrophyta	6926	Dictyochophyceae	269	Florenciellales	57	NA	0
Fragilariopsis	Fragilariopsis	5	Stramenopiles	10648	Ochrophyta	6926	Diatomeae	5430	Bacillariophyceae	1929	Fragilariopsis	135
Geminigera	Geminigera	7	Cryptophyceae	343	Cryptomonadales	439	Cryptomonadales	439	lineage-B	239	Geminigera	5
Haptolina	Haptolina	2	Haptophyta	953	Prymnesiophyceae	746	Prymnesiophyceae	746	Prymnesiaceae	59	Haptolina	7
Leptocylindrus	Leptocylindrus	1	Stramenopiles	10648	Ochrophyta	6926	Diatomeae	5430	Leptocylindrophyceae	53	Leptocylindrus	35
Mantoniella	Mantoniella	1	Chloroplastida	7436	Chlorophyta	7089	Mamiellophyceae	633	Mamiellaceae	68	Mantoniella	3
Micromonas	Micromonas	20	Chloroplastida	7436	Chlorophyta	7089	Mamiellophyceae	633	Mamiellaceae	68	Micromonas	44
Minidiscus	Minidiscus	2	Stramenopiles	10648	Ochrophyta	6926	Diatomeae	5430	Mediophyceae	2325	Minidiscus	5
Minutocellus	Minutocellus	2	Stramenopiles	10648	Ochrophyta	6926	Diatomeae	5430	Mediophyceae	2325	Minutocellus	4
Ostreococcus	Ostreococcus	4	Chloroplastida	7436	Chlorophyta	7089	Mamiellophyceae	633	Bathycoccaceae	19	Ostreococcus	14
Pelagococcus	Pelagococcus	2	Stramenopiles	10648	Ochrophyta	6926	Pelagophyceae	119	Pelagomonadales	84	Pelagococcus	14
Phaeocystis	Phaeocystis	27	Haptophyta	953	Prymnesiophyceae	746	Prymnesiophyceae	746	Phaeocystales	104	Phaeocystis	80
Picochlorum	Picochlorum	1	Chloroplastida	7436	Chlorophyta	7089	Trebouxiophyceae	879	Chlorellales	524	Picochlorum	4
Prasinoderma	Prasinoderma	2	Chloroplastida	7436	Palmophyllophyceae	37	Prasinoderma-clade	31	Prasinoderma-clade	31	Prasinoderma	21
Pseudo-nitzschia	Pseudo-nitzschia	7	Stramenopiles	10648	Ochrophyta	6926	Diatomeae	5430	Bacillariophyceae	1929	Pseudo-nitzschia	275
Pycnococcus	Pycnococcus	3	Chloroplastida	7436	Chlorophyta	7089	Pycnococcaceae	12	Pycnococcaceae	12	Pycnococcus	12
Skeletonema	Skeletonema	3	Stramenopiles	10648	Ochrophyta	6926	Diatomeae	5430	Mediophyceae	2325	Skeletonema	45
Strombidium	Strombidium	3	Alveolata	78782**	Ciliophora	18850	Spirotrichea	2121	Oligotrichia	1361	Sapolatum-clade	155
Synedropsis	Synedropsis	1	Stramenopiles	10648	Ochrophyta	6926	Diatomeae	5430	Bacillariophyceae	1929	Synedropsis	60
Thalassiosira	Thalassiosira	5	Stramenopiles	10648	Ochrophyta	6926	Diatomeae	5430	Mediophyceae	2325	Thalassiosira	593
unidentified Bacillariaceae 1	New_Bacillariaceae_01	3	Stramenopiles	10648	Ochrophyta	6926	Diatomeae	5430	NA	0	NA	0
unidentified Bacillariaceae 2	New_Bacillariaceae_02	2	Stramenopiles	10648	Ochrophyta	6926	Diatomeae	5430	NA	0	NA	0
unidentified Bacillariaceae 3	Unidentified_Bacillariaceae	2	Stramenopiles	10648	Ochrophyta	6926	Diatomeae	5430	NA	0	NA	0
unidentified Bicosoecida	New_Bicosoecales_01	8	Stramenopiles	10648	Opalozoa	1228	Bicosoecida	230	NA	0	NA	0
unidentified Cercozoa	Unidentified_Cercozoa	2	Rhizaria	19891	Cercozoa	2625	NA	0	NA	0	NA	0
unidentified Chlorophyta	New_Chlorophyta_01	6	Chloroplastida	7436	Chlorophyta	7089	NA	0	NA	0	NA	0
unidentified Choanoflagellatea	New_Choanozoa_01	11	Opisthokonta	33545**	Choanoflagellata	298	NA	0	NA	0	NA	0
unidentified Choreotrichida	New_Choreotrichida _01	15	Alveolata	78782**	Ciliophora	18850	Spirotrichea	2121	Oligotrichia	1361	NA	0
unidentified Chrysochromulinaceae	New_Chrysochromulinaceae_01	39	Haptophyta	953	Prymnesiophyceae	746	Prymnesiophyceae	746	Chrysochromulinaceae	333	NA	0
unidentified Chrysophyceae	New_Chrysophyceae	23	Stramenopiles	10648	Ochrophyta	6926	Chrysophyceae	397	NA	0	NA	0
unidentified Ciliophora	New_Ciliophora_01	2	Alveolata	78782**	Ciliophora	18850	NA	0	NA	0	NA	0
unidentified Cyrtolophosidida	New_Cyrtolophosidida_01	4	Alveolata	78782**	Ciliophora	18850	Colpodea	182	Cyrtolophosidida	69	NA	0
unidentified Haptophyta	New_Haptophyta_01	10	Haptophyta	953	NA	0	NA	0	NA	0	NA	0
unidentified MALV-I 1	New_MALV-I_01	2	Alveolata	78782**	Dinoflagellata	55005**	MALV-I	11911	NA	0	NA	0
unidentified MALV-I 2	MALV-I	2	Alveolata	78782**	Dinoflagellata	55005**	MALV-I	11911	NA	0	NA	0
unidentified MALV-II 1	New_MALV-II_01	2	Alveolata	78782**	Dinoflagellata	55005**	MALV-II	9698	NA	0	NA	0
unidentified MALV-II 2	MALV-II	3	Alveolata	78782**	Dinoflagellata	55005**	MALV-II	9698	NA	0	NA	0
unidentified MAST-04	New_MAST-4	25	Stramenopiles	10648	Sagenista	1655	MAST-04	68	NA	0	NA	0
unidentified Pelagomonadales	Sister_Pelagomonas	2	Stramenopiles	10648	Ochrophyta	6926	Pelagophyceae	119	Pelagomonadales	84	Pelagomonas	8
