# Supplementary material for: Diverse patterns of correspondence between protist metabarcodes and protist metagenome-assembled genomes
Source: PLoS One. 2024 Jun 6;19(6):e0303697. doi: 10.1371/journal.pone.0303697 (PMC11156365; doi:10.1371/journal.pone.0303697)
Supplement: S2 File — (ZIP) [file pone.0303697.s002.zip › S2_table.pdf]

Genome taxonomy	Genome/SMAG ID	GenBank18S ID	Scaffold with 18S	Tara V9 barcode	Tara V9 blast % identity
Cafeteria roenbergensis	VLTN01000011.1 / TARA_ION_45_MAG_00147	MN334557	GCA_008330645.1_CrBVI_genomic	dedd73012459fedb2d854754f2c4acf248d1712d	100
Pycnococcus provasolii	OW568862.1 / TARA_AON_82_MAG_00189	X91264	GCA_938743325.1_ucPycProv1.1_genomic	e637558ec51a7fe0a2cb11d338d2079bc7d74786	100
Ostreococcus lucimarinus	NC_009366.1 / TARA_AON_82_MAG_00012	Y15814	GCF_000092065.1_ASM9206v1_genomic	6b514fe39086fcf7c1325878394f32b5e6141e95	100
Micromonas pusilla	NW_003315882.1 / TARA_AON_82_MAG_00118	KT860843	GCF_000151265.2_Micromonas_pusilla_CCMP1545_v2.0_genomic	495f75dd678a6700fd8f3fc3fab2aa87800d0aa1	100
unidentified Chrysophyceae	Metagenome_centric_SAG_TOSAG00_8	Z38025	Metagenome_centric_SAG_TOSAG00_8_scaffold23	920df2f681bb3553916becceea25586702322ecd	99.231
unidentified Bicosoecida_01	Metagenome_centric_SAG_TOSAG00_9	EF023971	Metagenome_centric_SAG_TOSAG00_9_scaffold260	aca17e422ca891974c6af687ccf37be1e685b732	100
unidentified Chrysophyceae	Metagenome_centric_SAG_TOSAG23_30	Z38025	Metagenome_centric_SAG_TOSAG23_30_scaffold1	d945a94b464aed8e5322f7fd0a04bd30f6355957	100
unidentified Bacillariaceae 3	Metagenome_centric_SAG_TOSAG39_4	Y10570	Metagenome_centric_SAG_TOSAG39_4_scaffold39	c9ed411fc2cb7c5b69ca7d739c193c9bad6af9bd	100
unidentified Chrysophyceae	Metagenome_centric_SAG_TOSAG41_5	Z38025	Metagenome_centric_SAG_TOSAG41_5_scaffold18	1eb8e3f6b2b77f89775fe68f7693c1deb0a1a299	100
unidentified Bacillariaceae 1	Metagenome_centric_SAG_TOSAG46_2	Y10570	Metagenome_centric_SAG_TOSAG46_2_scaffold29	a642e178c5f32337ab5f4d18c97ae6cb32d93dd9	100
