# Supplementary material for: Diverse patterns of correspondence between protist metabarcodes and protist metagenome-assembled genomes
Source: PLoS One. 2024 Jun 6;19(6):e0303697. doi: 10.1371/journal.pone.0303697 (PMC11156365; doi:10.1371/journal.pone.0303697)
Supplement: S2 File — (ZIP) [file pone.0303697.s002.zip › S8_fig.pdf]

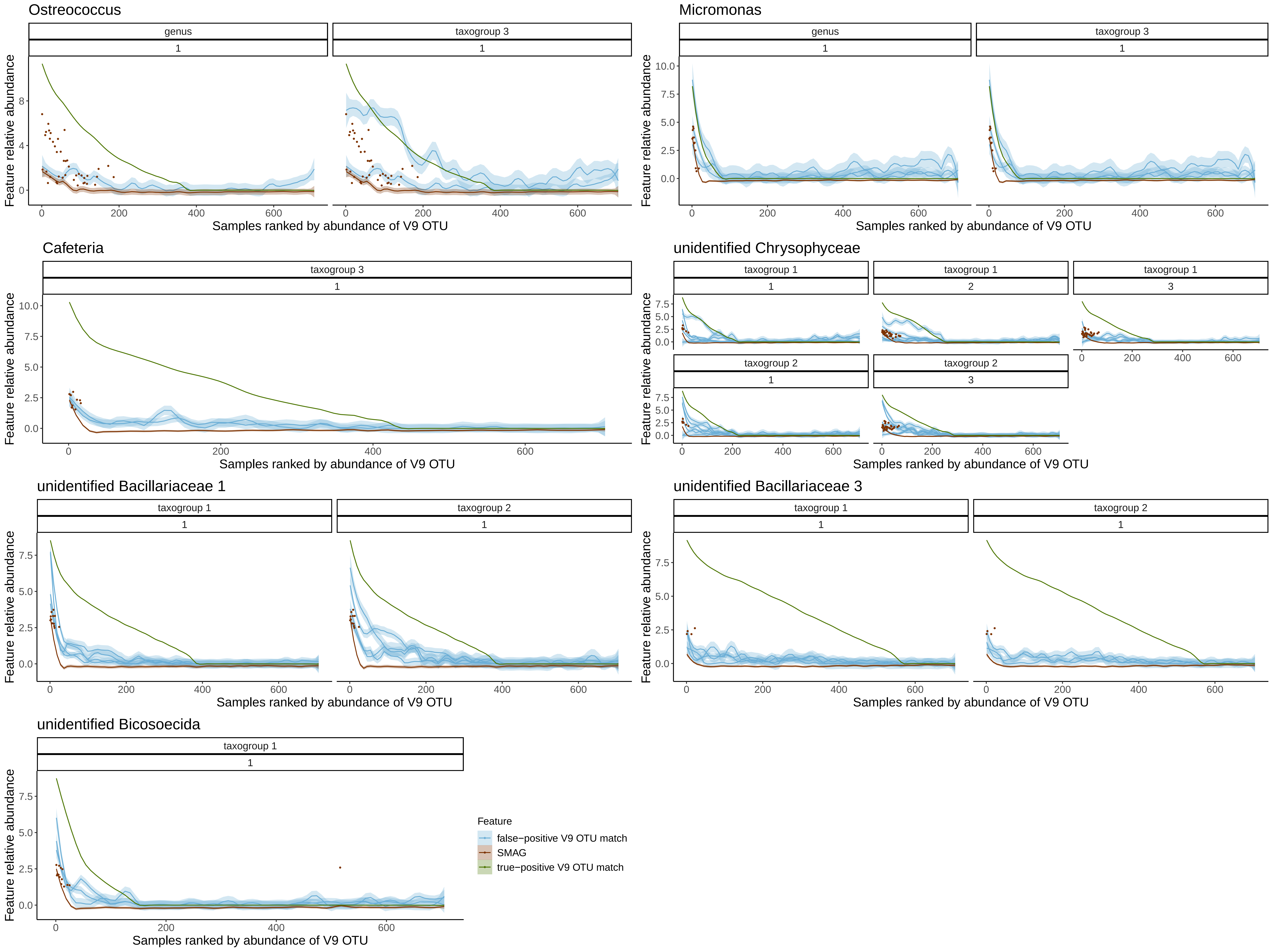
Ostreococcus
genus
1
taxogroup 3
1
Feature relative abundance
Samples ranked by abundance of V9 OTU
Micromonas
genus
1
taxogroup 3
1
Feature relative abundance
Samples ranked by abundance of V9 OTU
Cafeteria
taxogroup 3
1
Feature relative abundance
Samples ranked by abundance of V9 OTU
unidentified Chrysophyceae
taxogroup 1
1
taxogroup 1
2
taxogroup 1
3
taxogroup 2
1
taxogroup 2
3
Feature relative abundance
Samples ranked by abundance of V9 OTU
unidentified Bacillariaceae 1
taxogroup 1
1
taxogroup 2
1
Feature relative abundance
Samples ranked by abundance of V9 OTU
unidentified Bacillariaceae 3
taxogroup 1
1
taxogroup 2
1
Feature relative abundance
Samples ranked by abundance of V9 OTU
unidentified Bicosoecida
taxogroup 1
1
Feature relative abundance
Samples ranked by abundance of V9 OTU
Feature
false−positive V9 OTU match
SMAG
true−positive V9 OTU match
