# Supplementary figures and images for: Diverse patterns of correspondence between protist metabarcodes and protist metagenome-assembled genomes
Source: PLoS One. 2024 Jun 6;19(6):e0303697. doi: 10.1371/journal.pone.0303697 (PMC11156365; doi:10.1371/journal.pone.0303697)

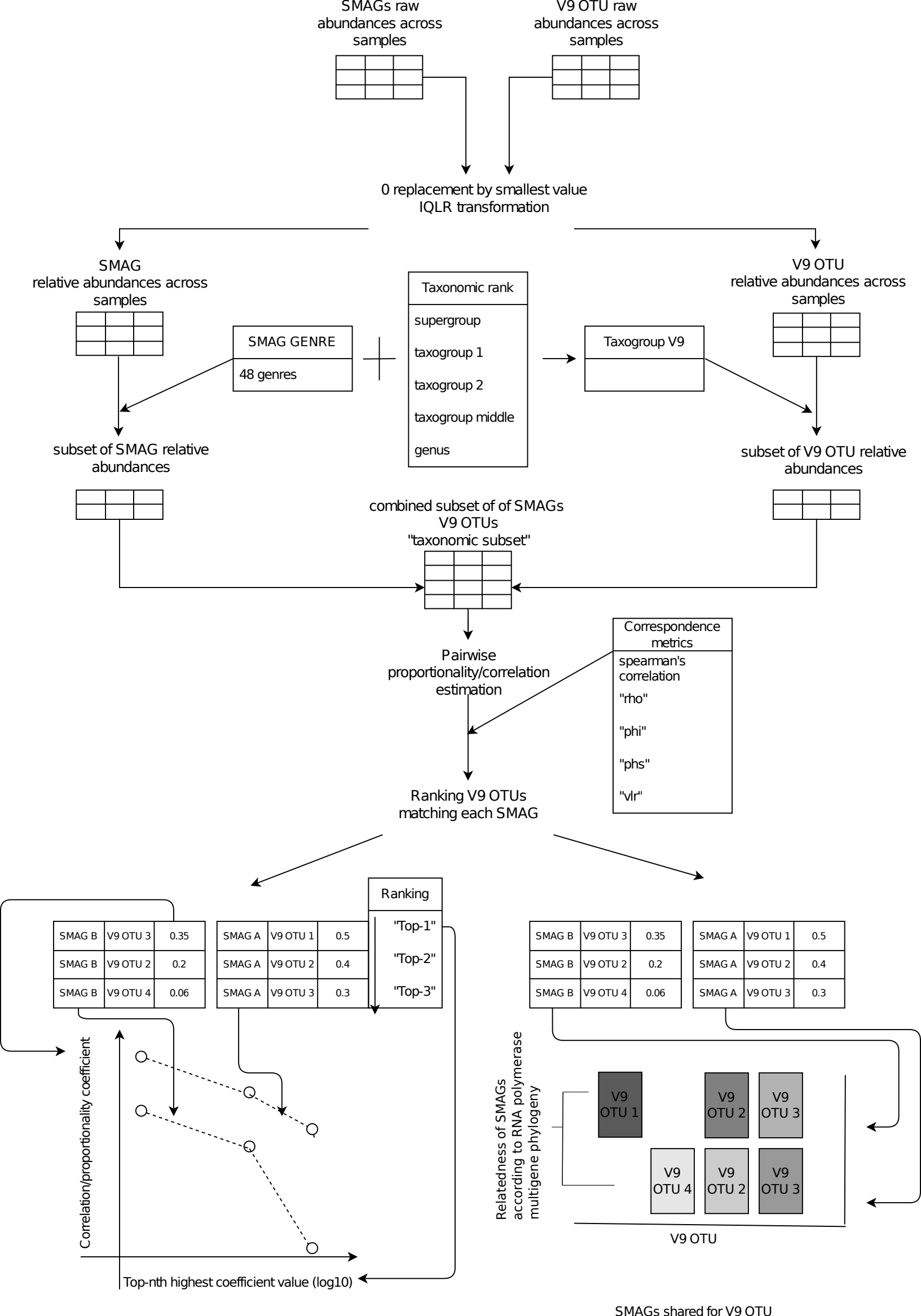

Supplement: S2 File — (ZIP) [file pone.0303697.s002.zip › S1_fig.pdf]

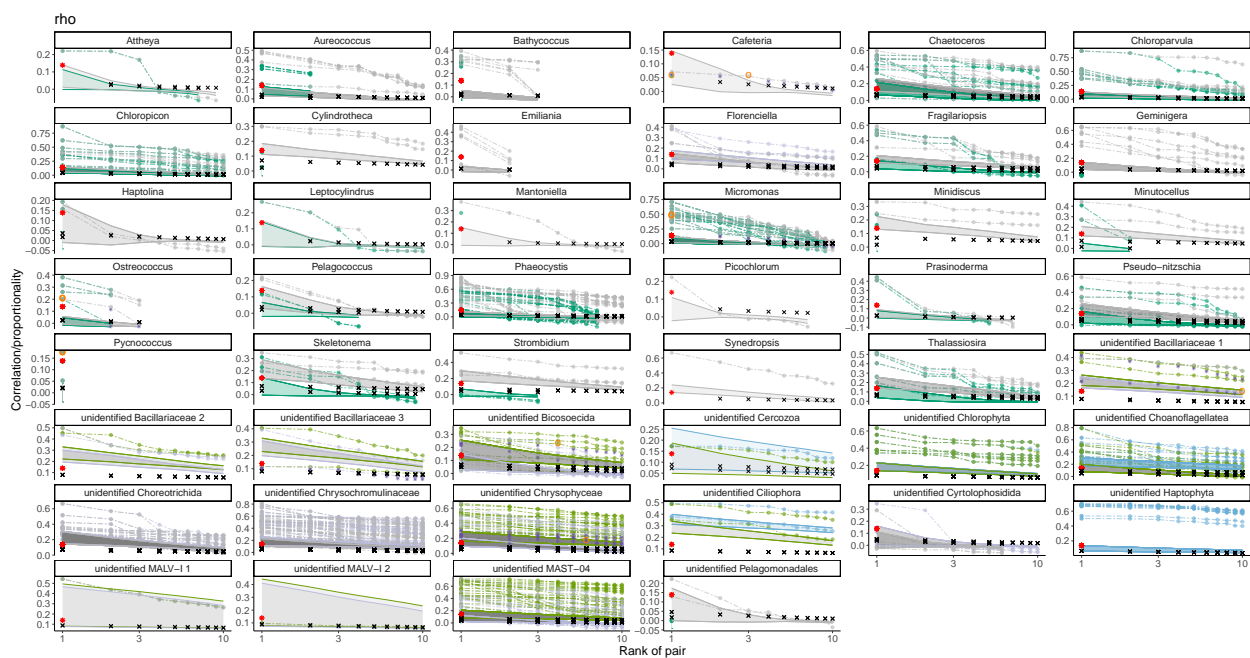

Figure S2: A

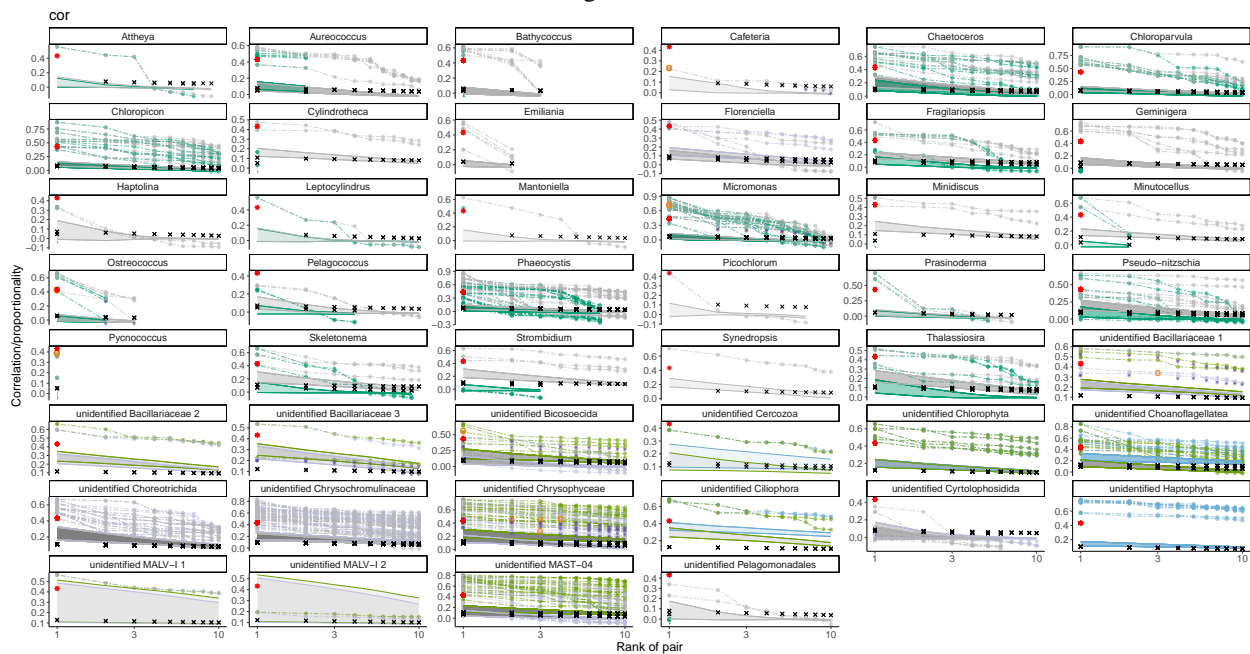

Figure S2: B

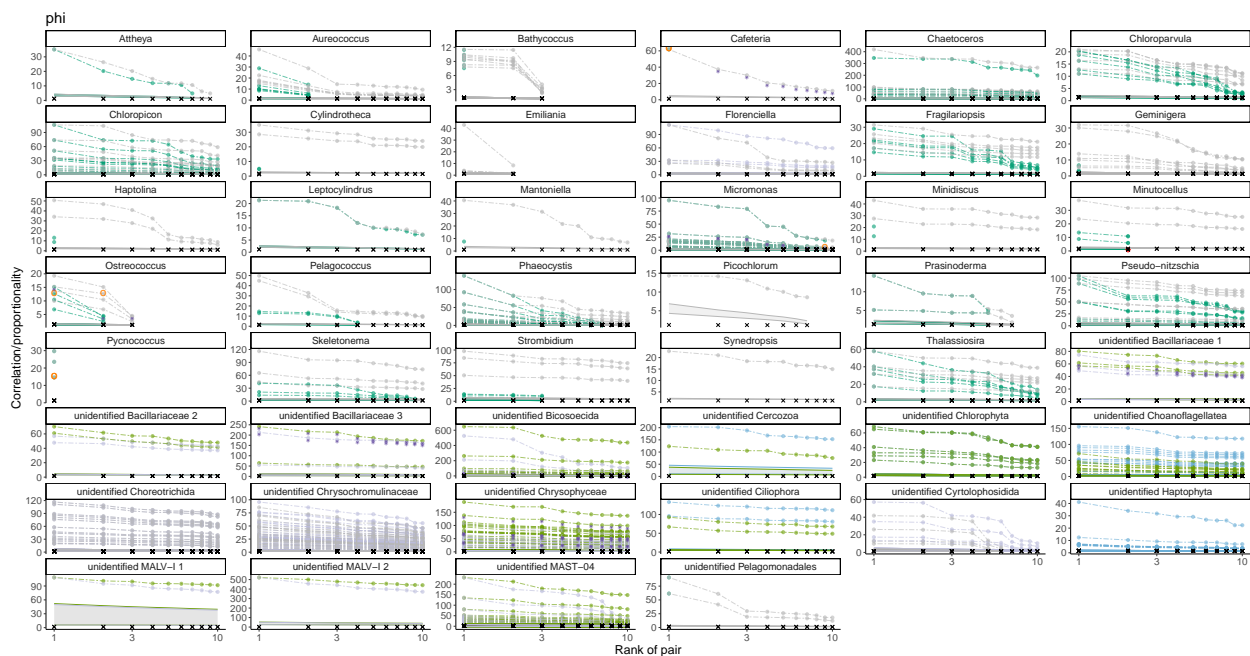

Figure S2: C

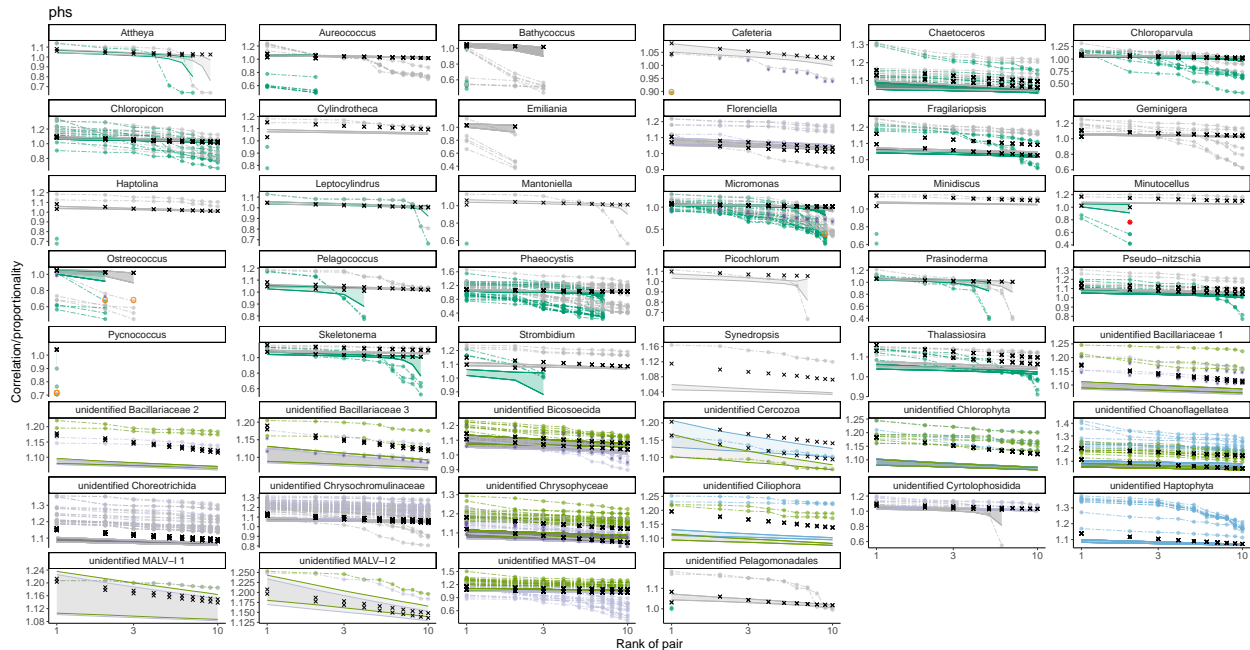

Figure S2: D

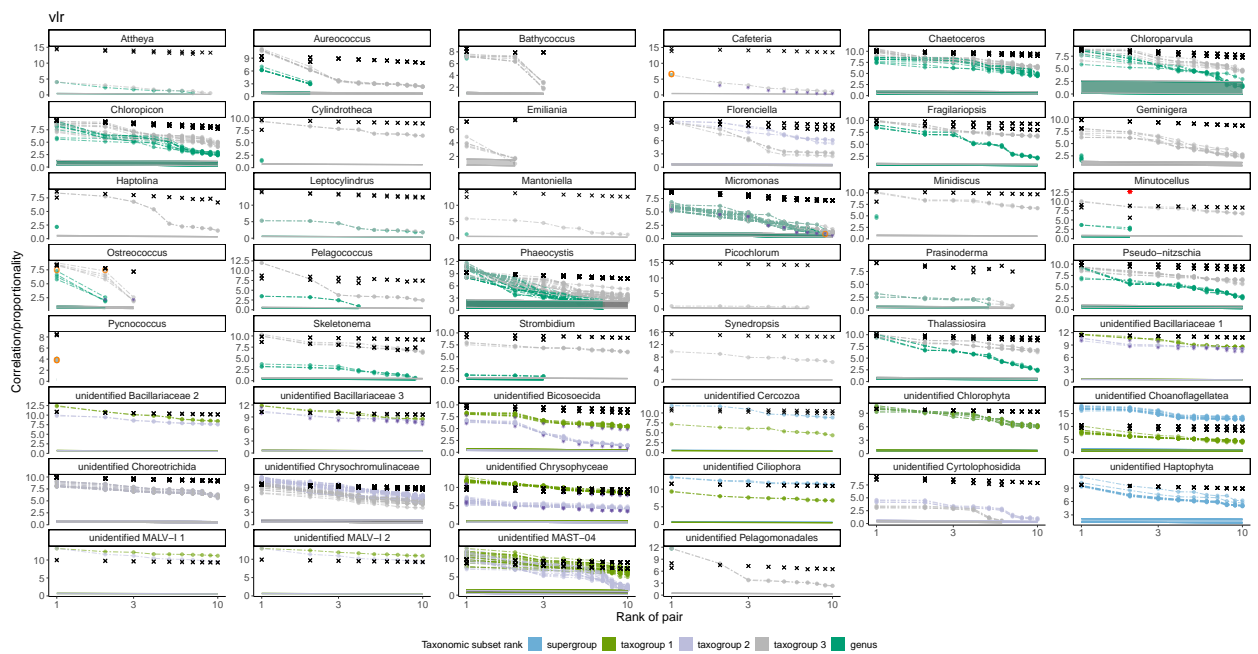

Figure S2: E

Supplement: S2 File — (ZIP) [file pone.0303697.s002.zip › S2_fig.pdf]

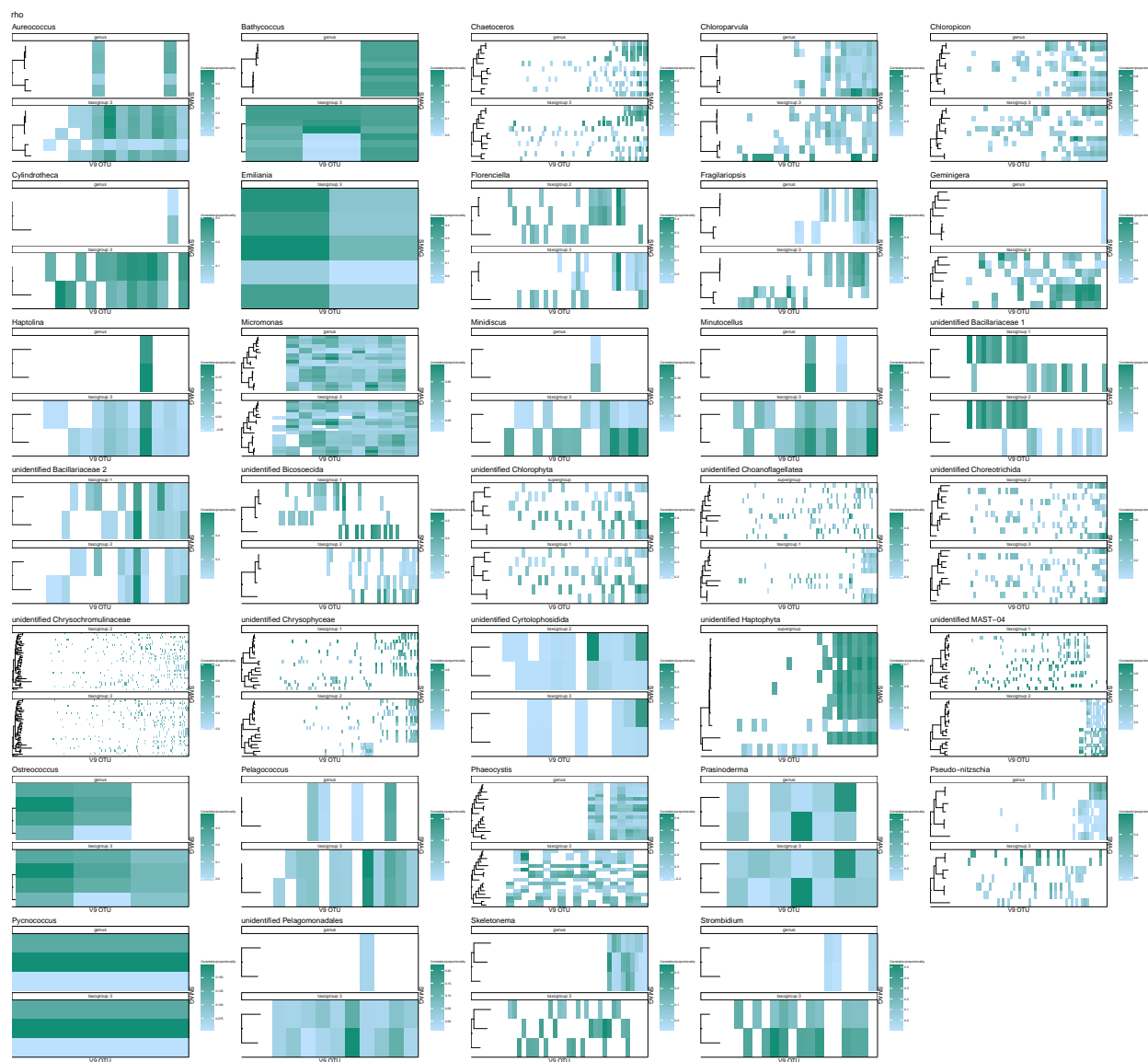

Figure S3: A

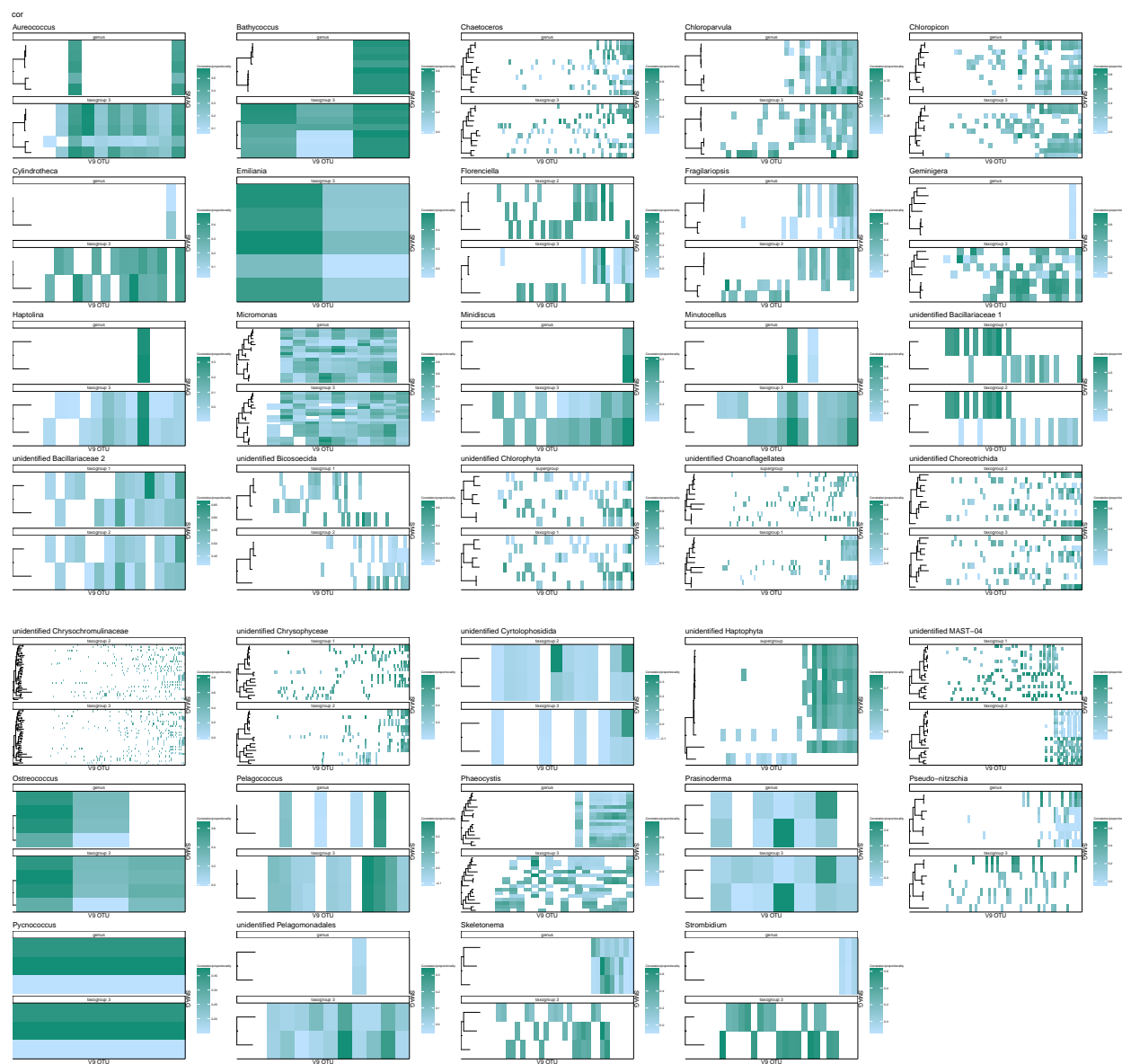

Figure S3: B

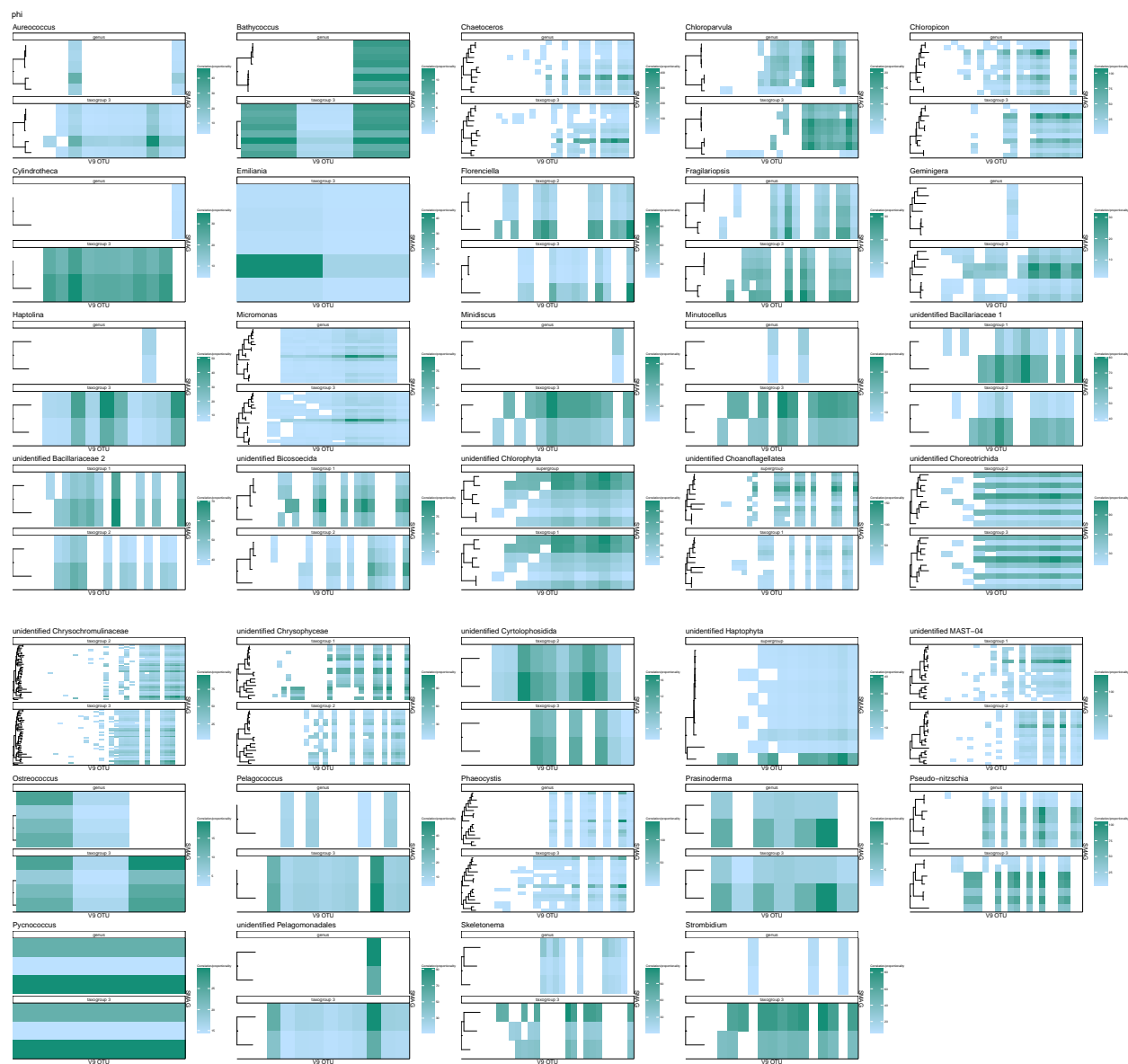

Figure S3: C

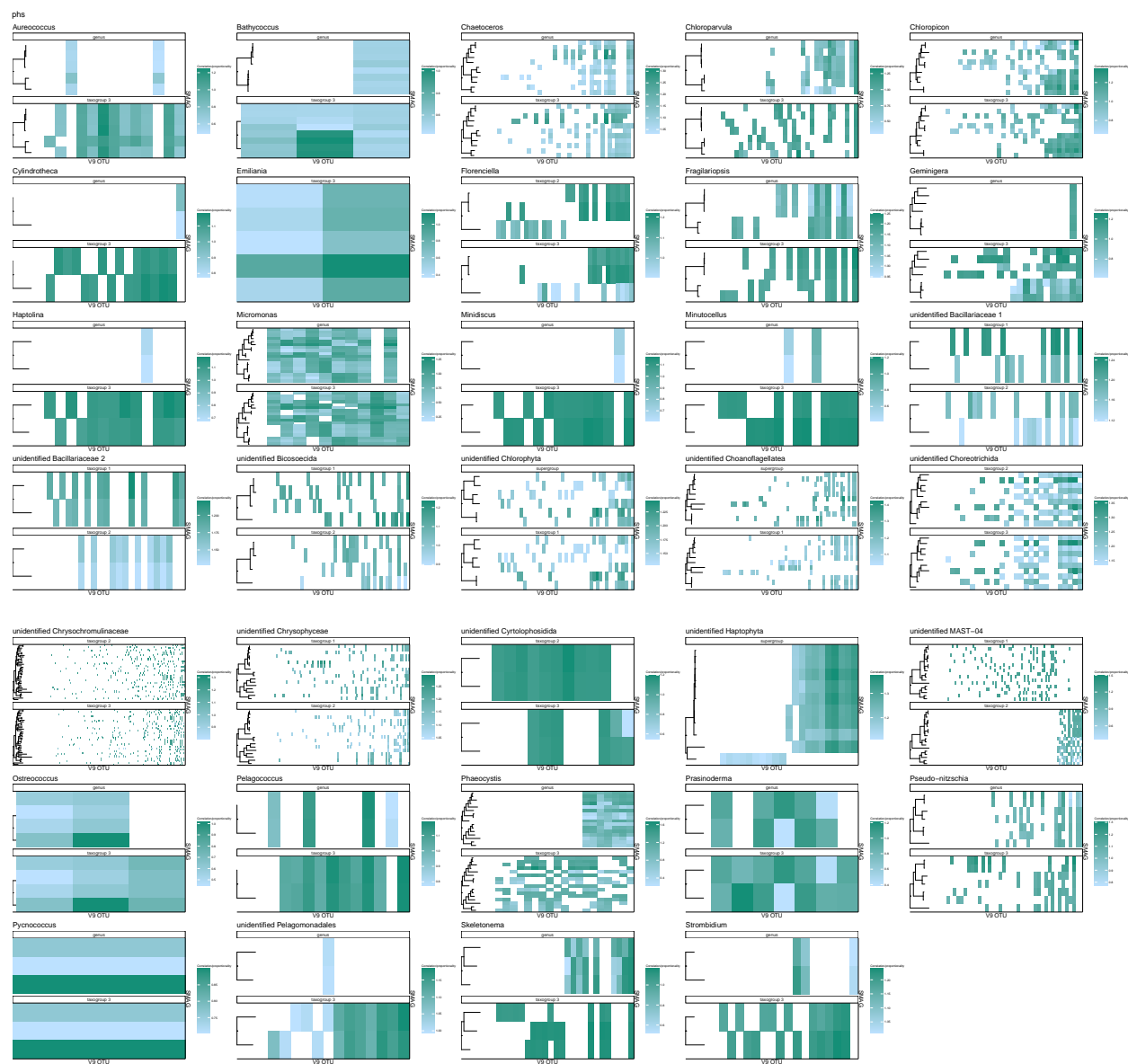

Figure S3: D

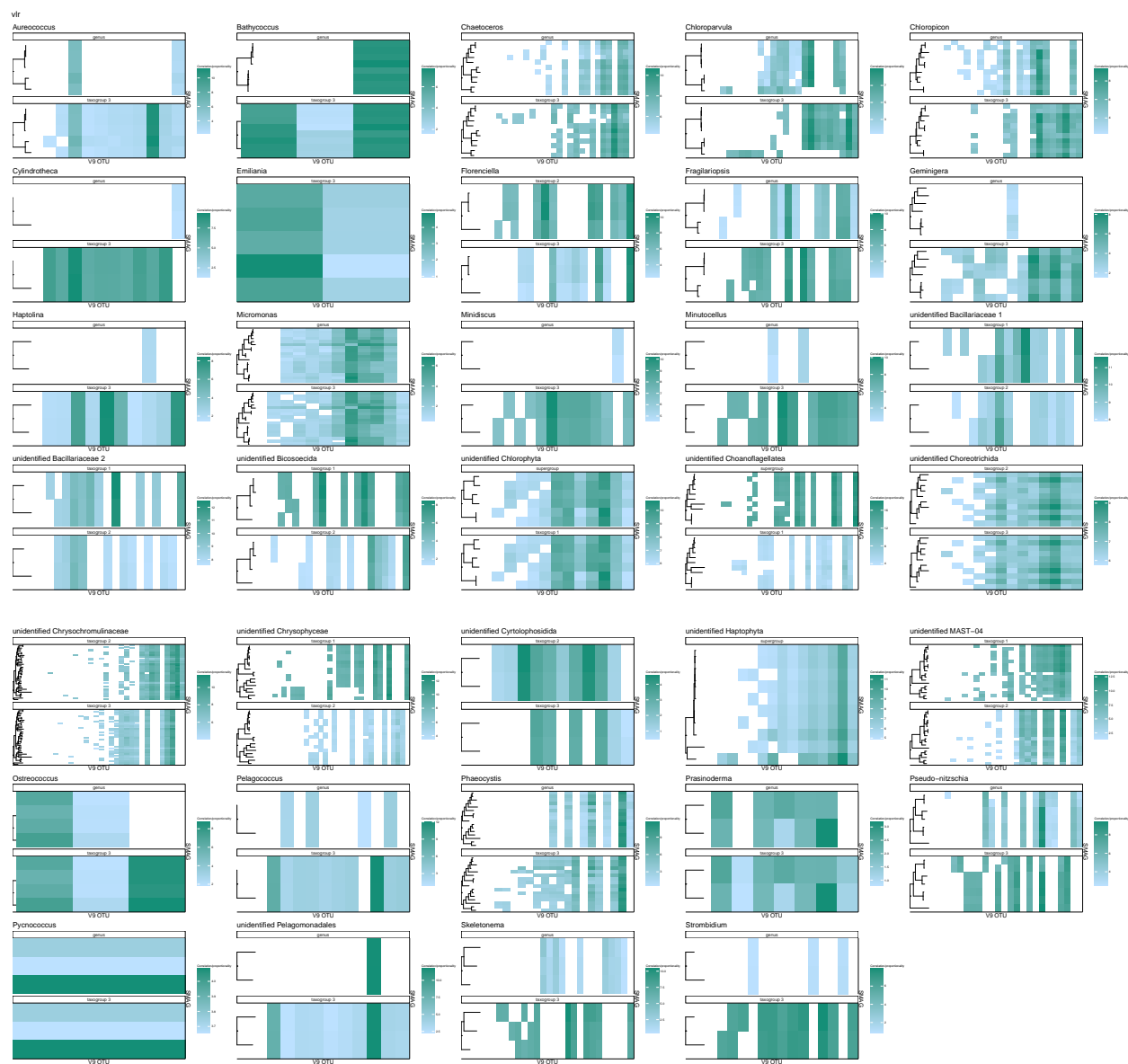

Figure S3: E

Supplement: S2 File — (ZIP) [file pone.0303697.s002.zip › S3_fig.pdf]

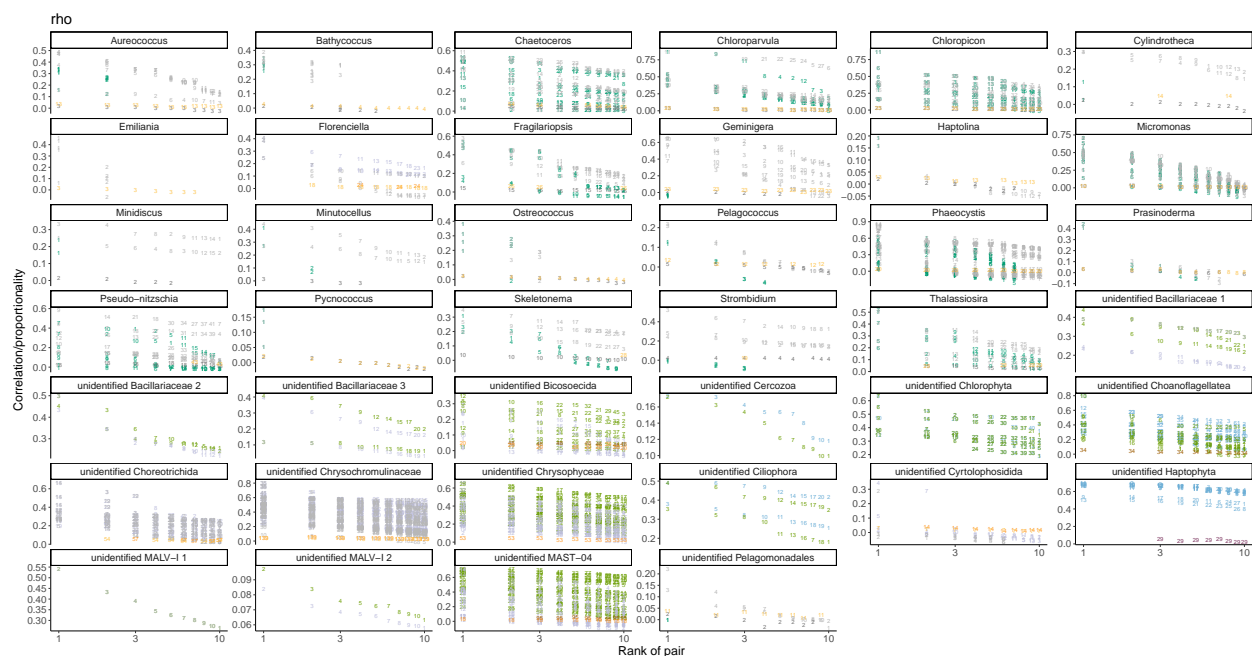

Figure S4: A

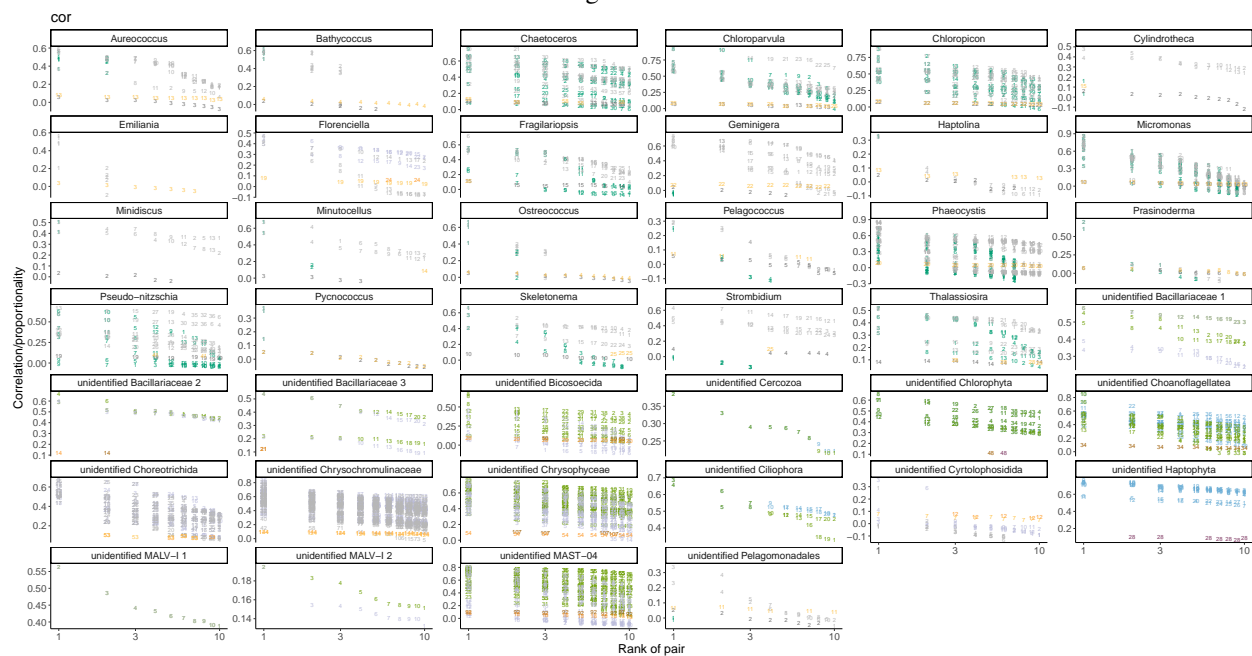

Figure S4: B

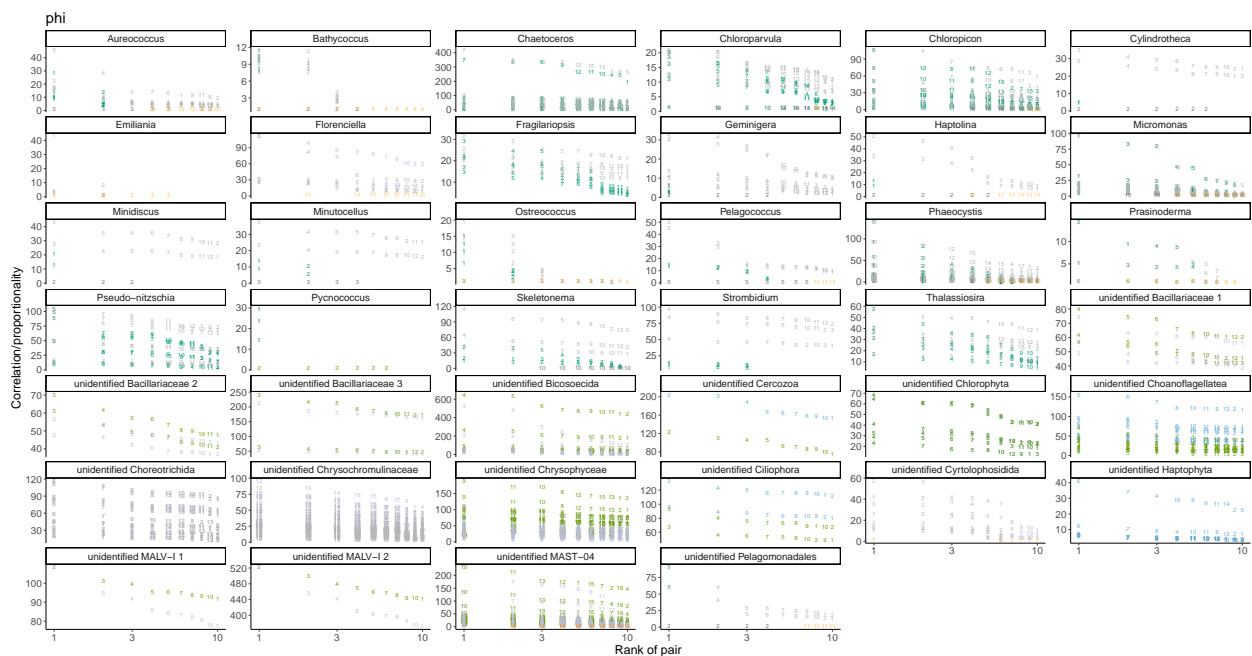

Figure S4: C

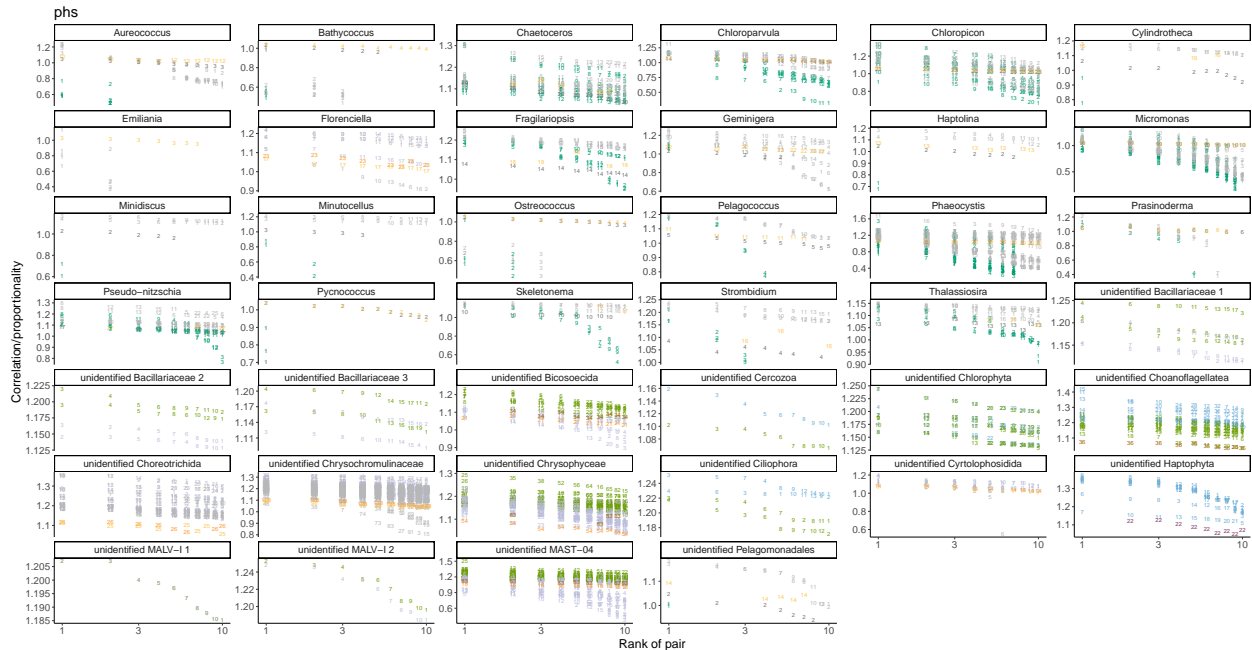

Figure S4: D

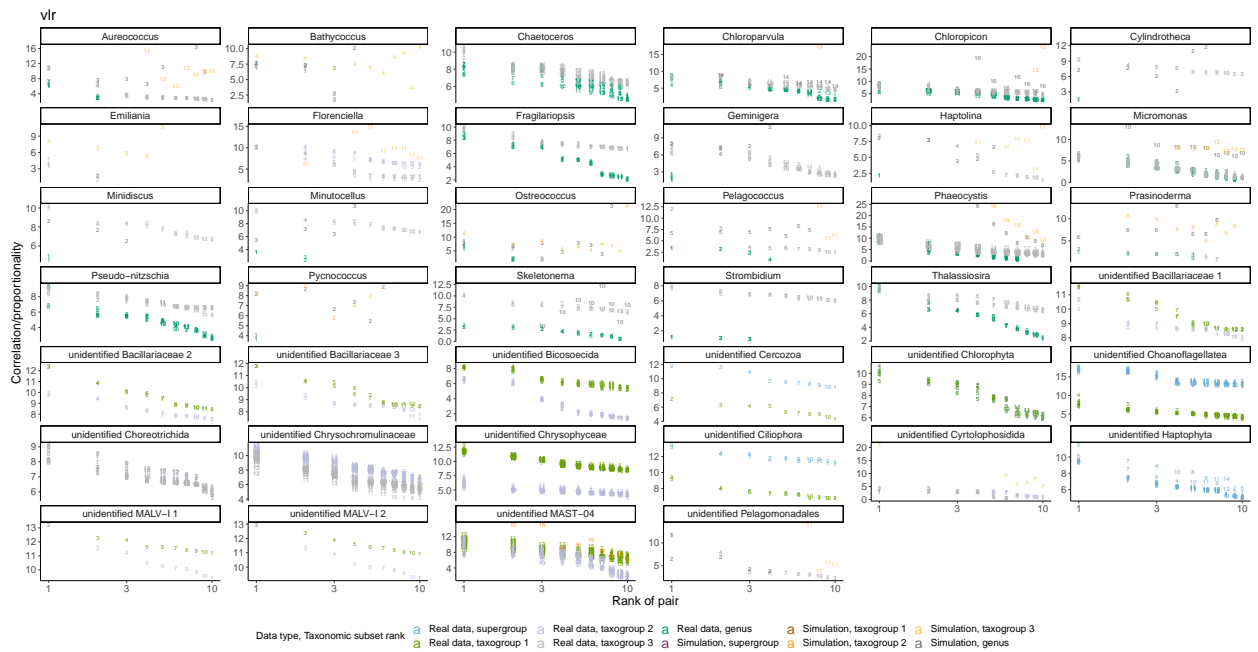

Figure S4: E

Supplement: S2 File — (ZIP) [file pone.0303697.s002.zip › S4_fig.pdf]

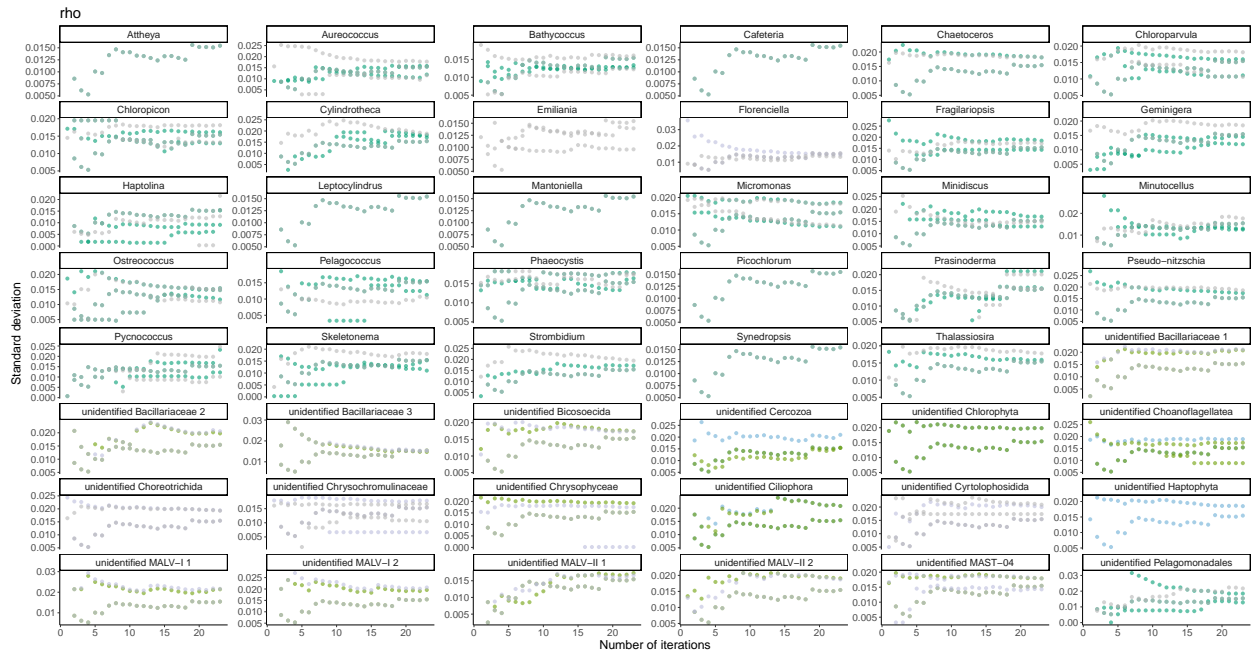

Figure S5: A

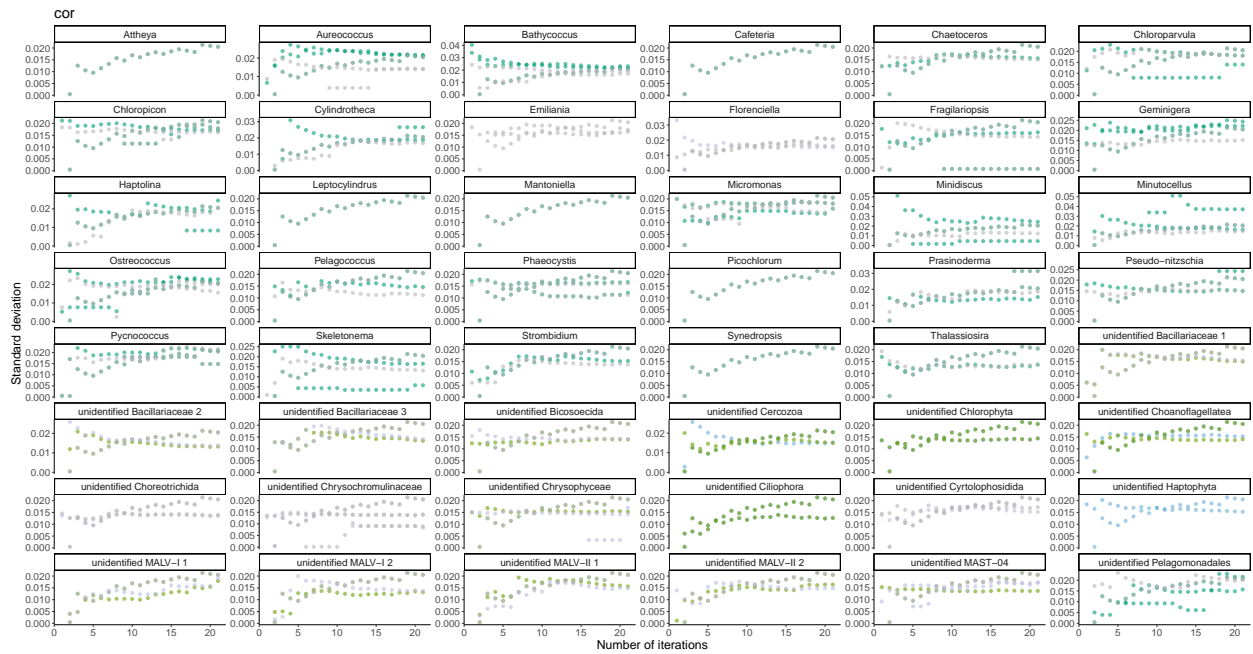

Figure S5: B

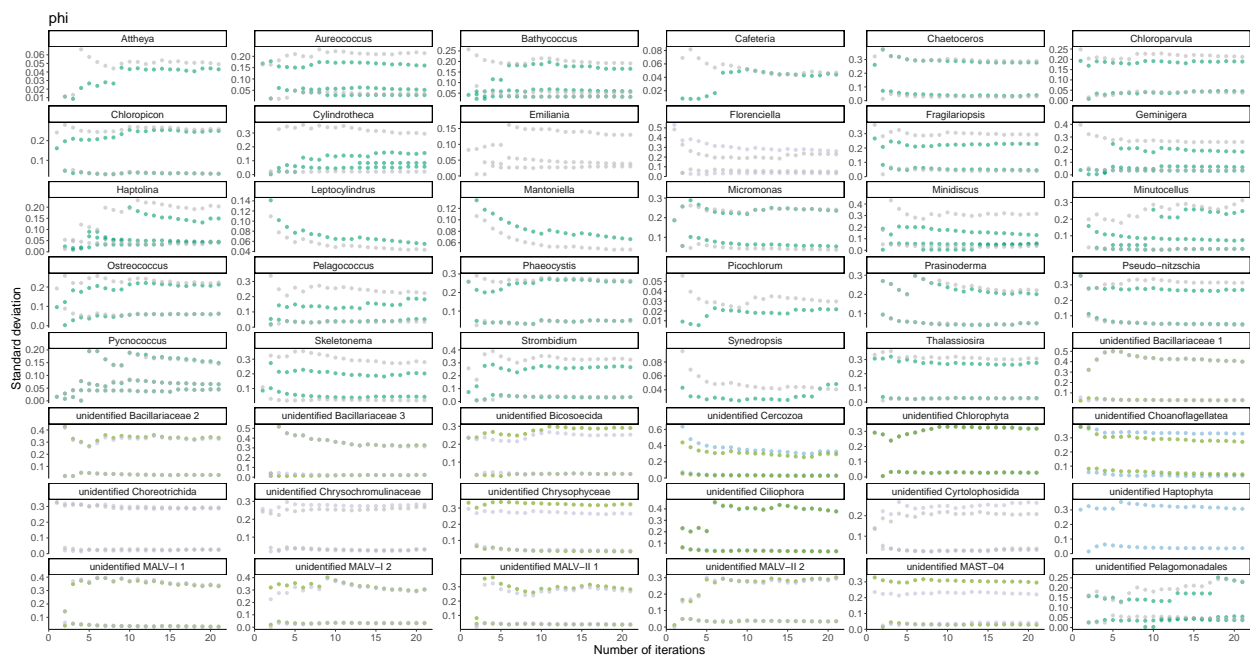

Figure S5: C

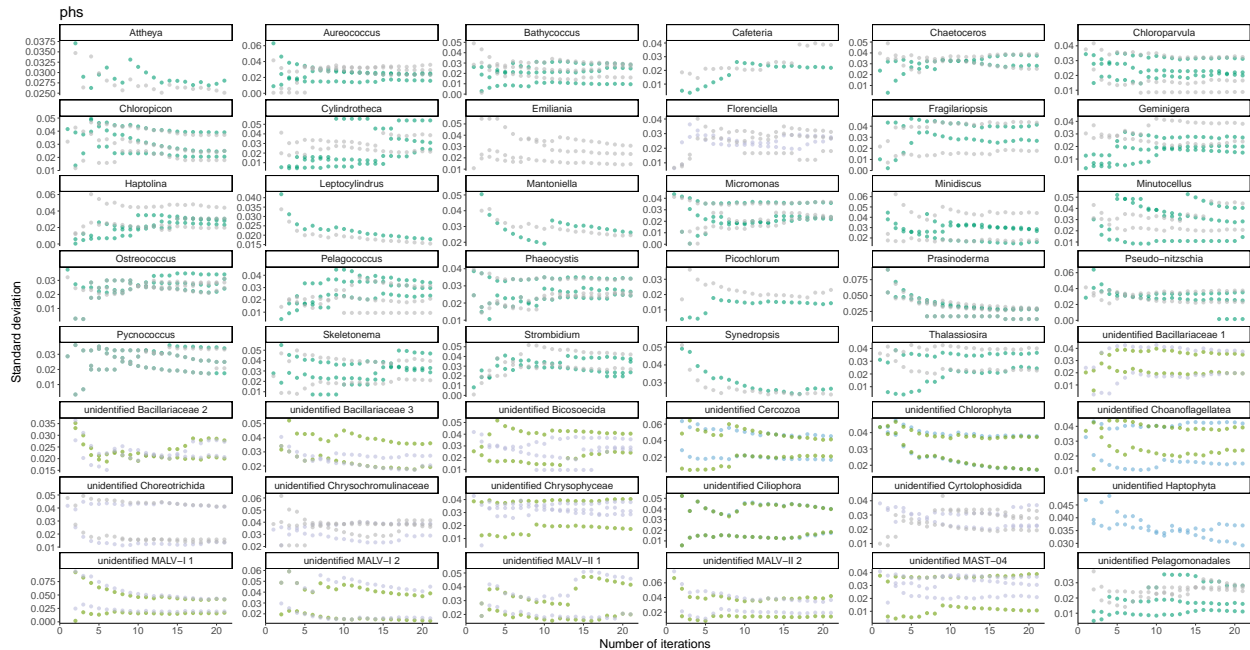

Figure S5: D

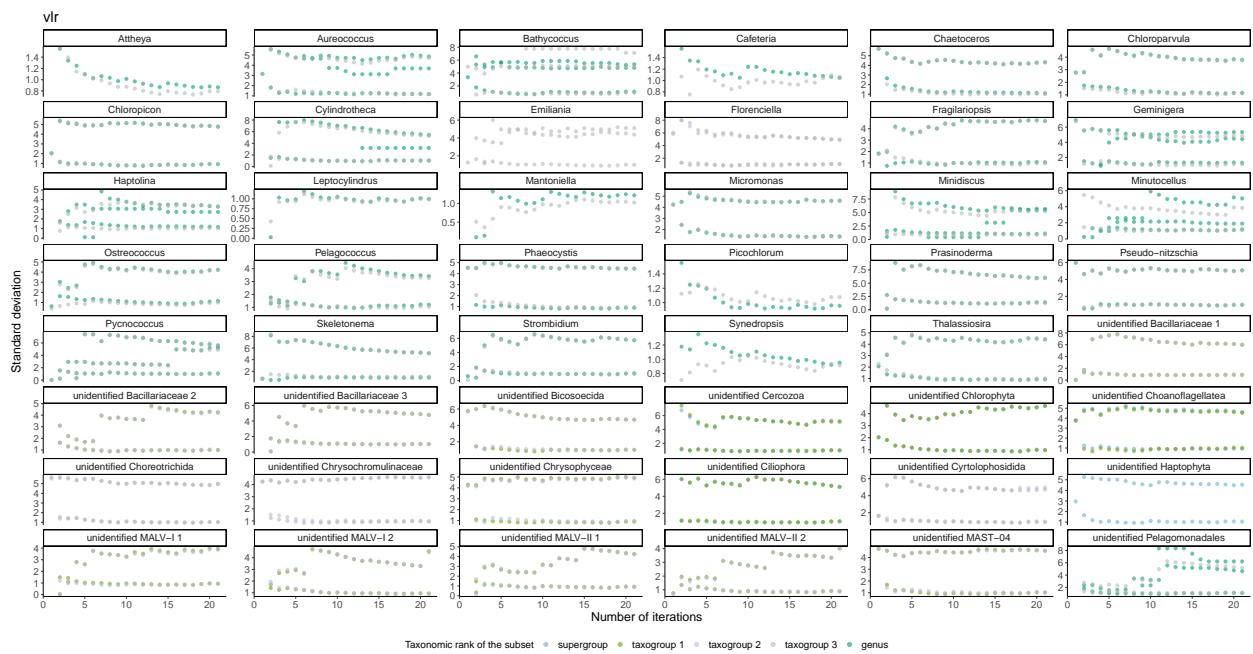

Figure S5: E

Supplement: S2 File — (ZIP) [file pone.0303697.s002.zip › S5_fig.pdf]

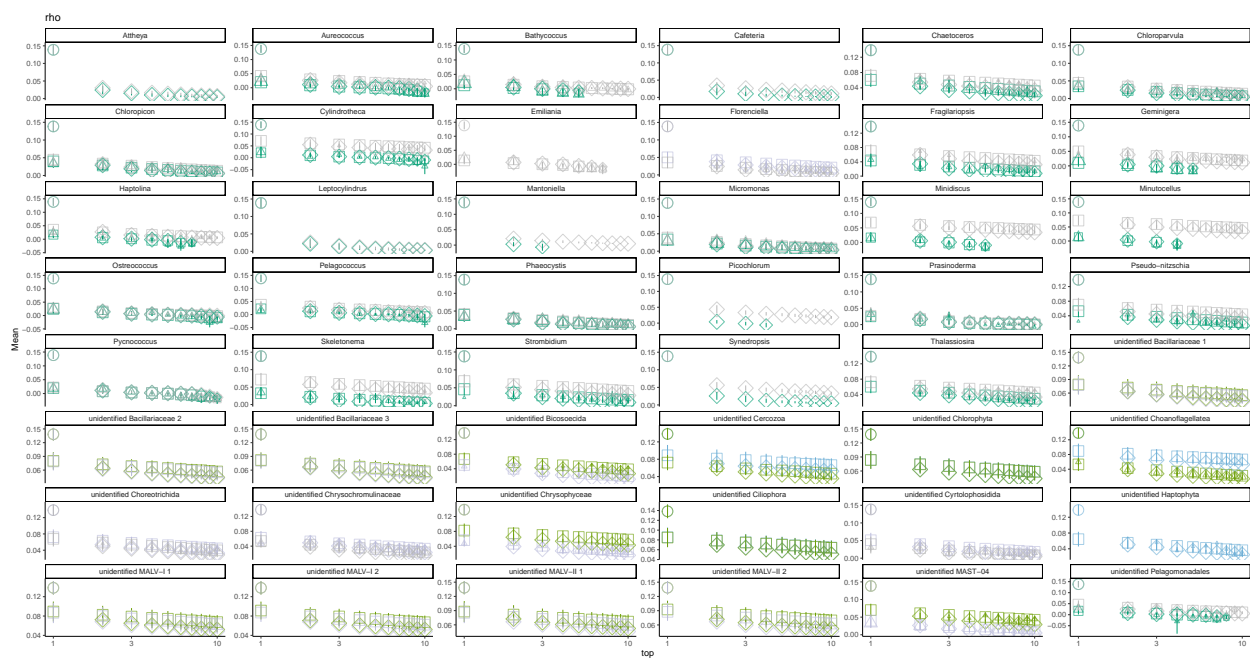

Figure S6: A

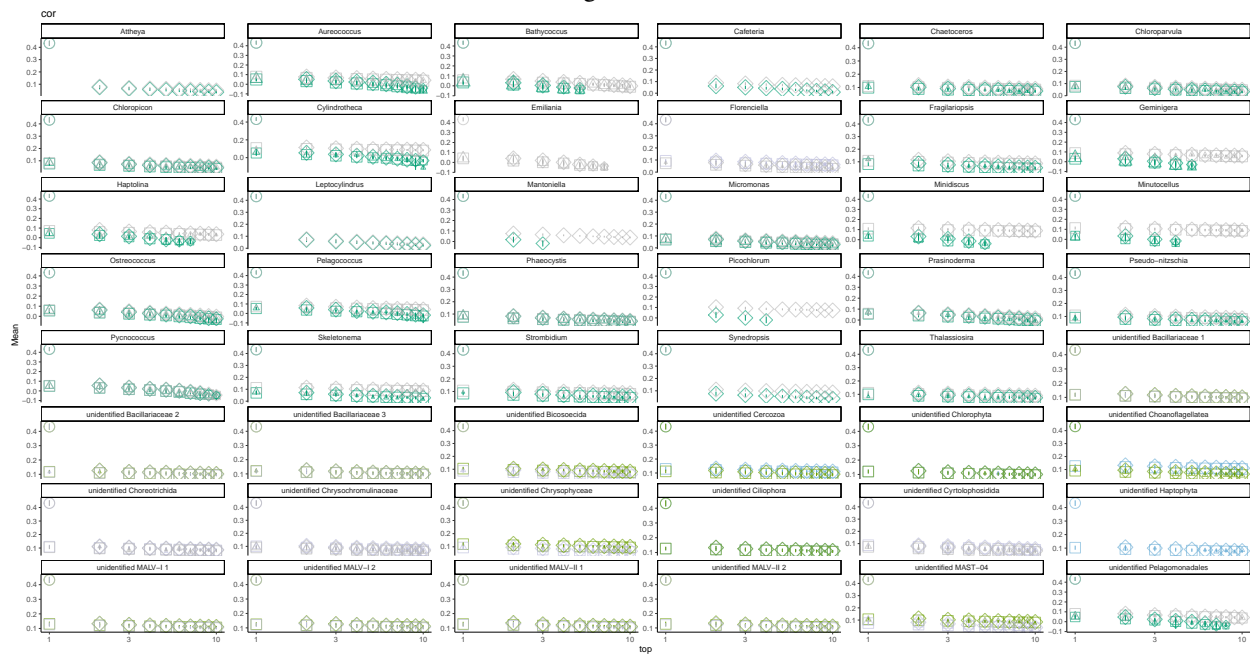

Figure S6: B

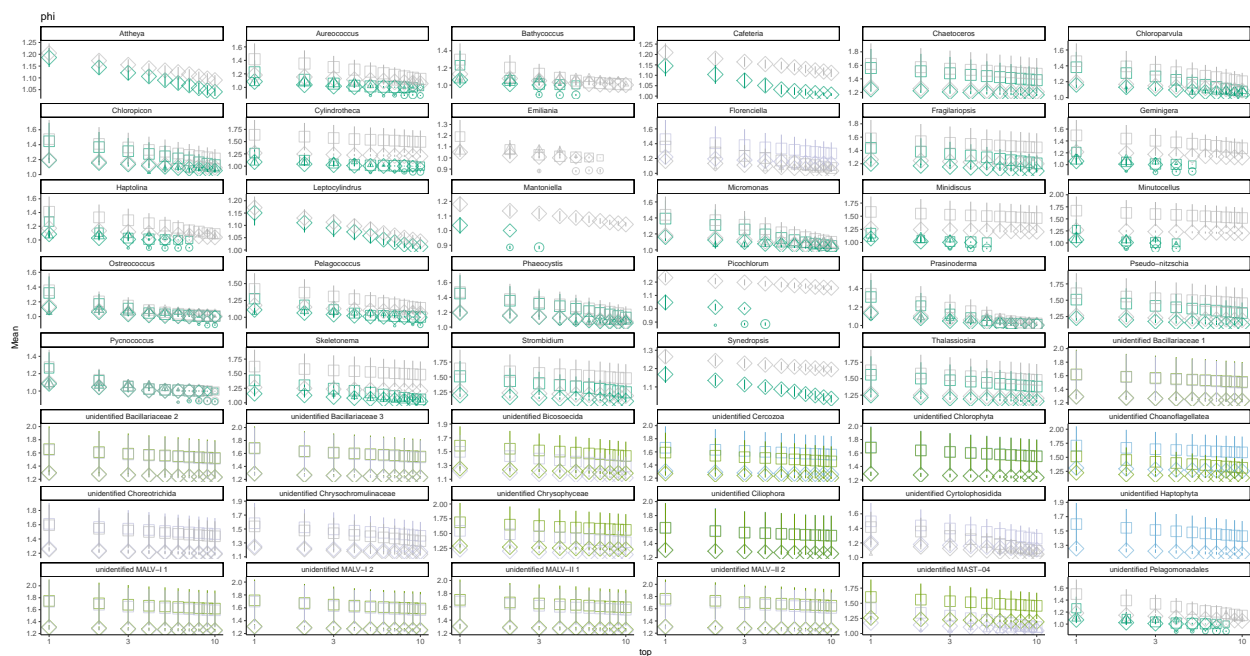

Figure S6: C

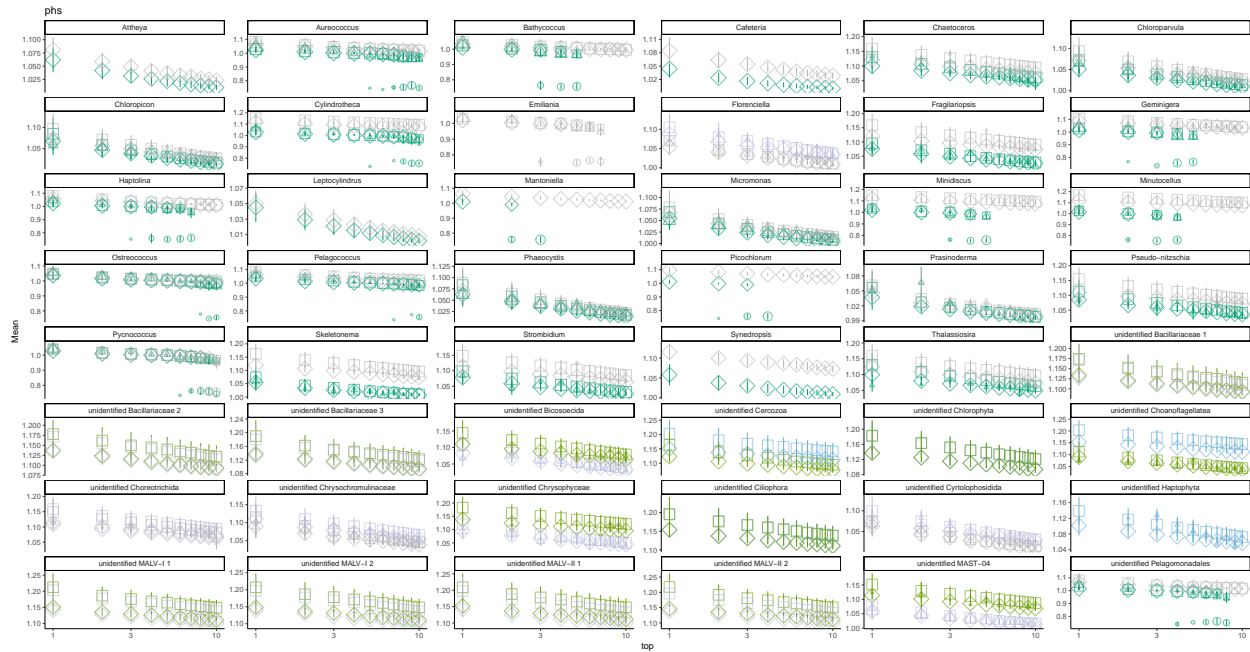

Figure S6: D

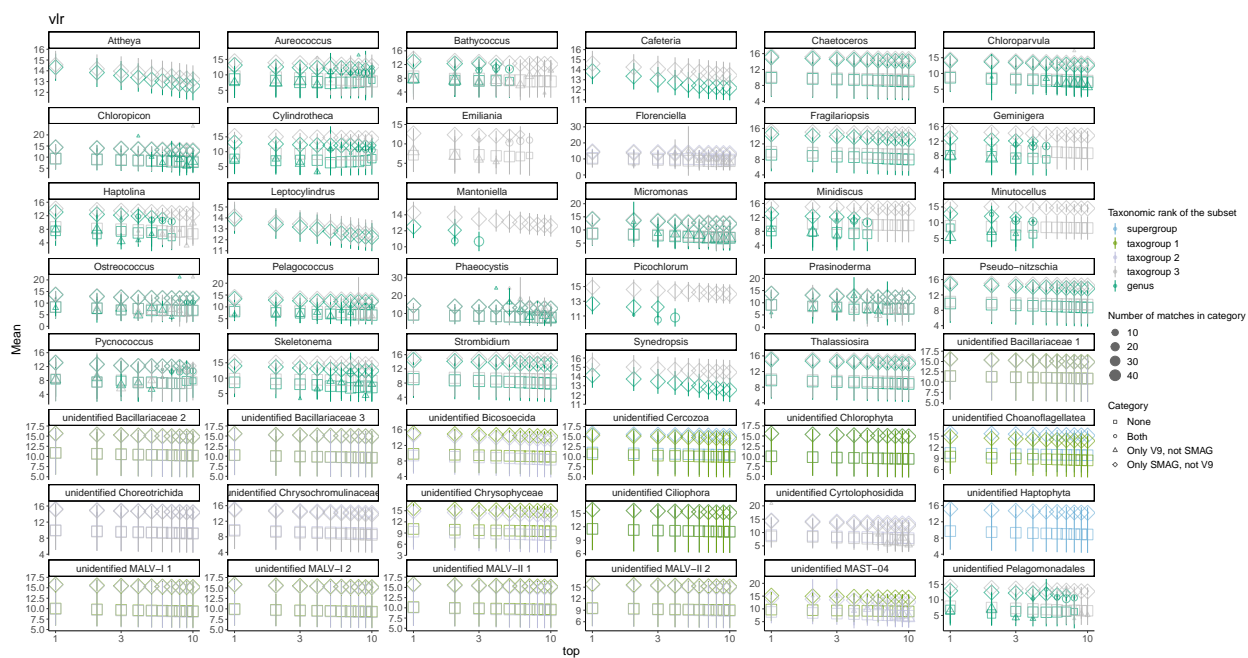

Figure S6: E

Supplement: S2 File — (ZIP) [file pone.0303697.s002.zip › S6_fig.pdf]

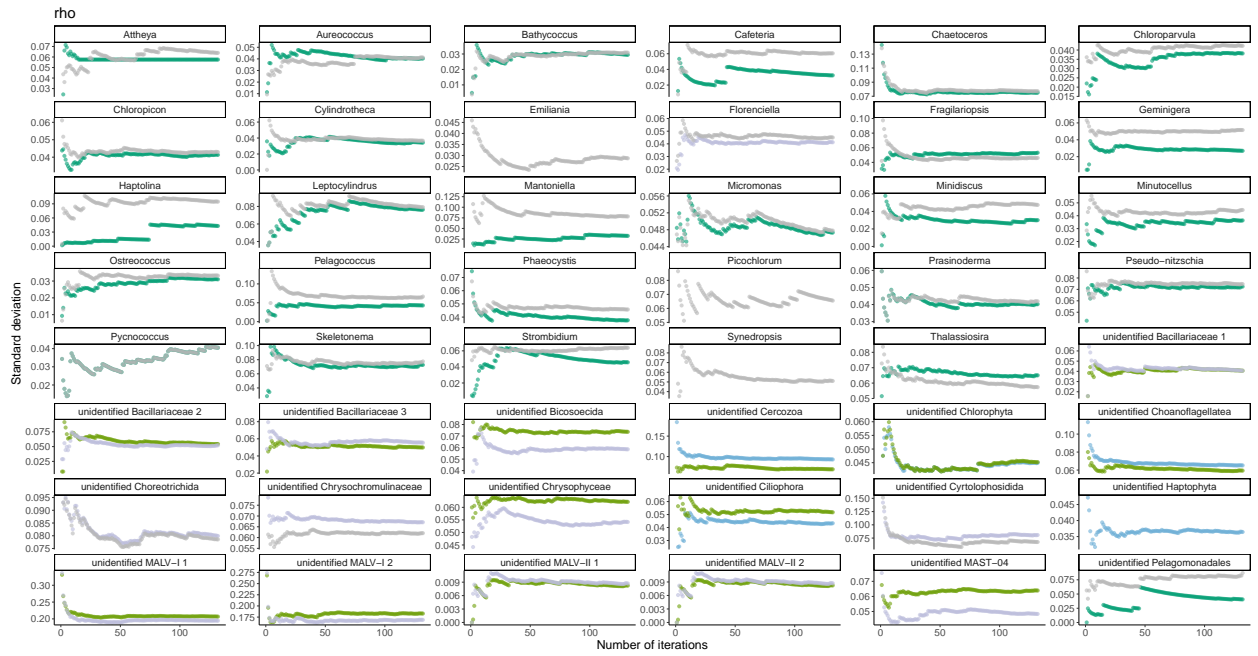

Figure S7: A

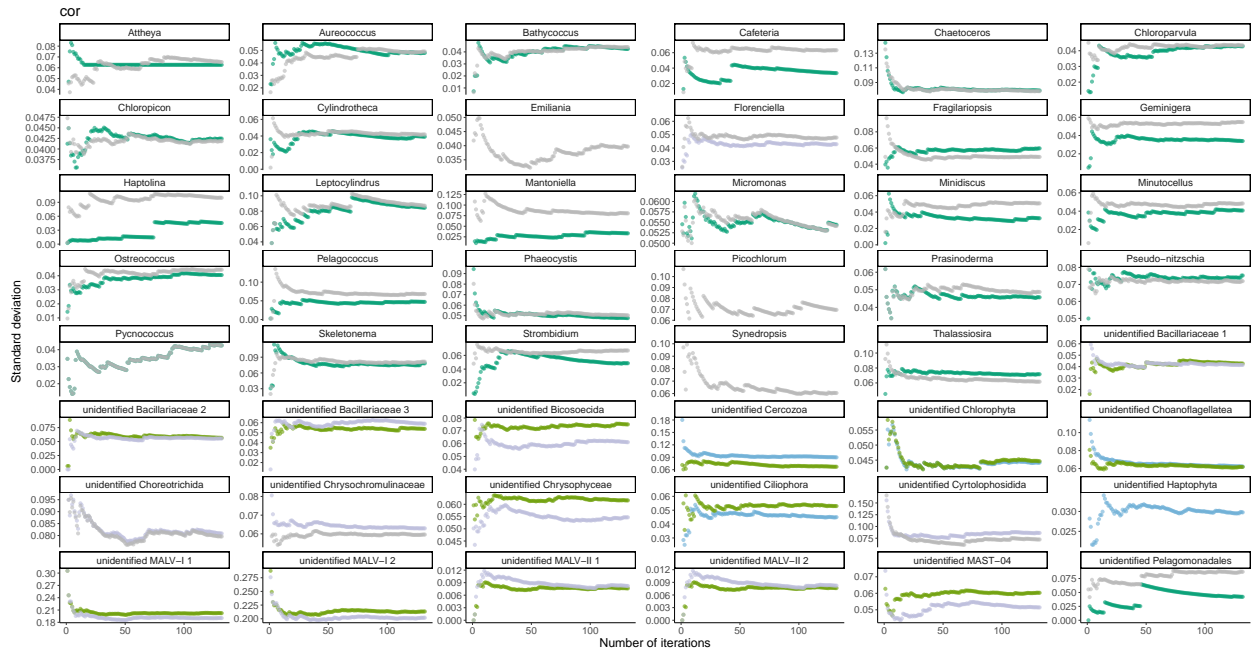

Figure S7: B

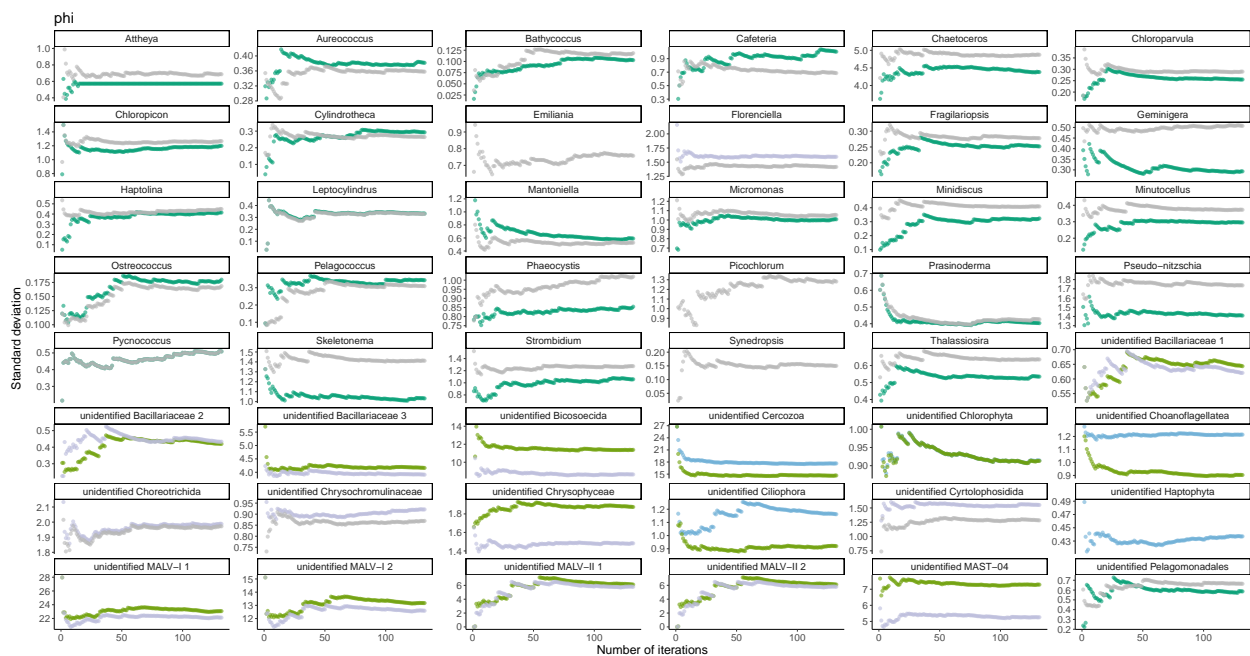

Figure S7: C

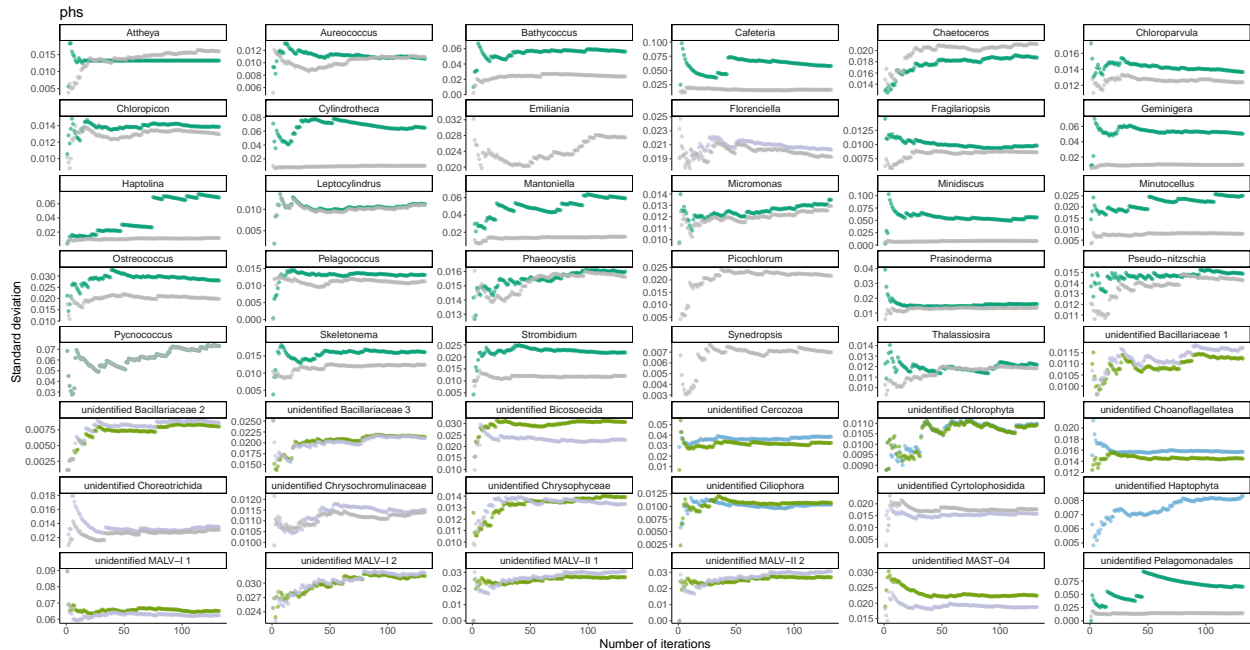

Figure S7: D

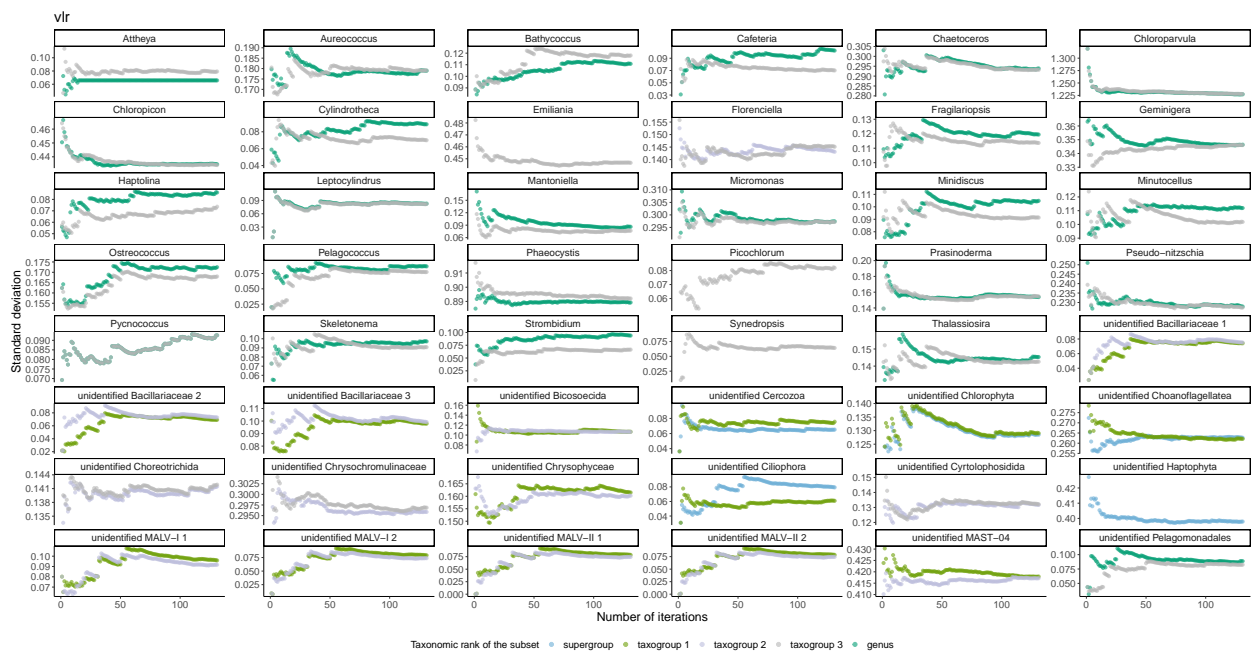

Figure S7: E

Supplement: S2 File — (ZIP) [file pone.0303697.s002.zip › S7_fig.pdf]

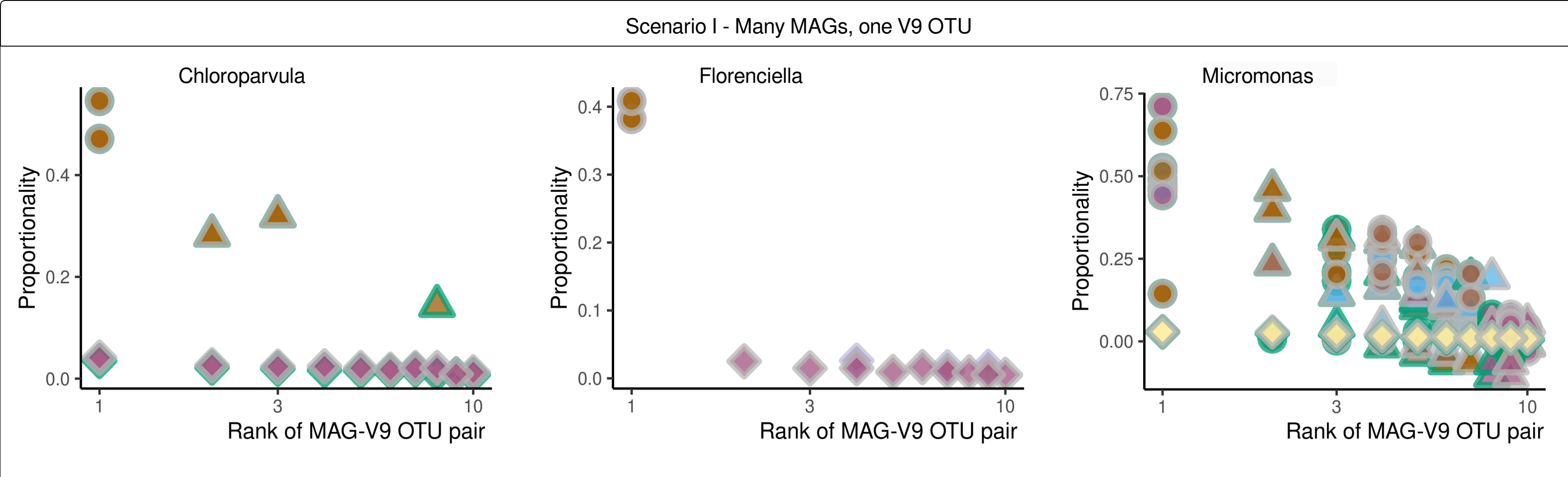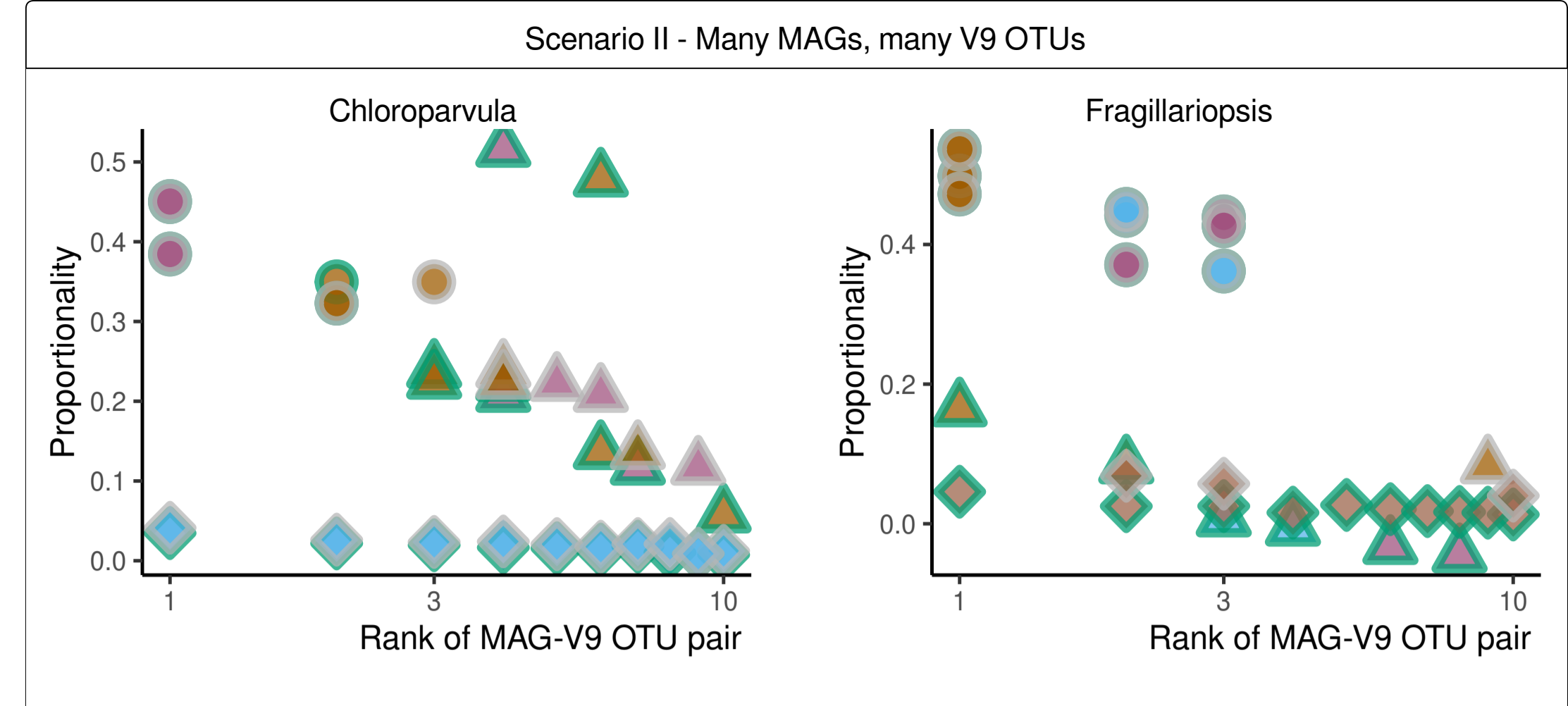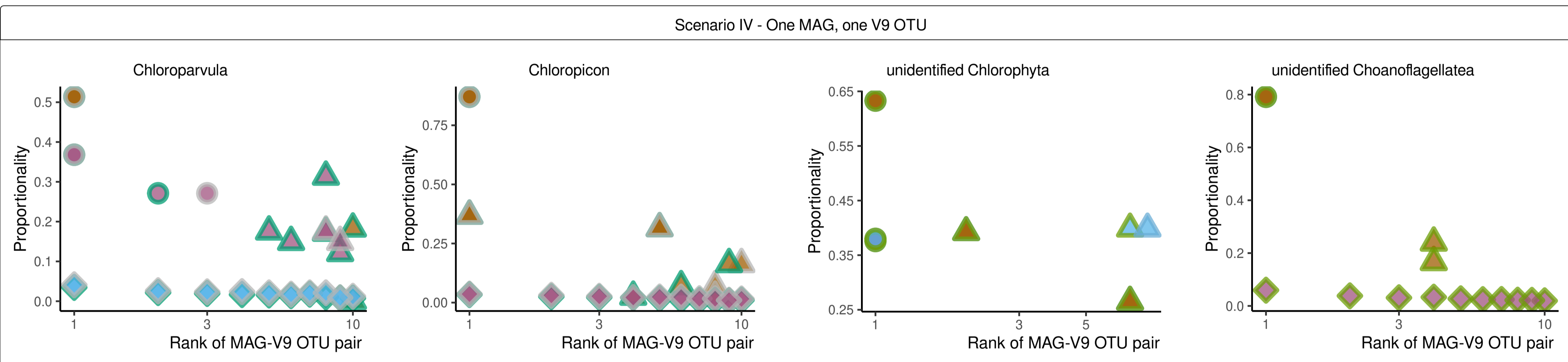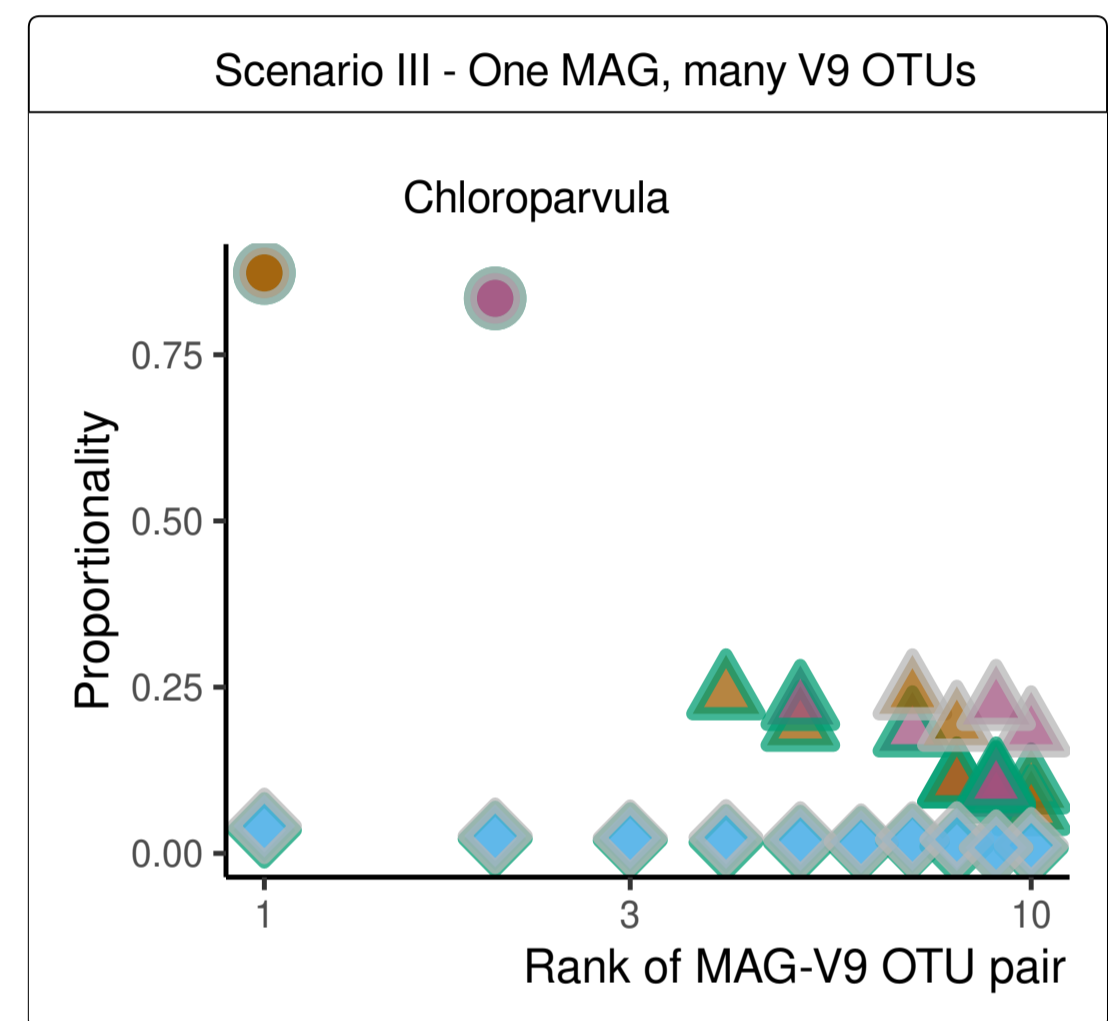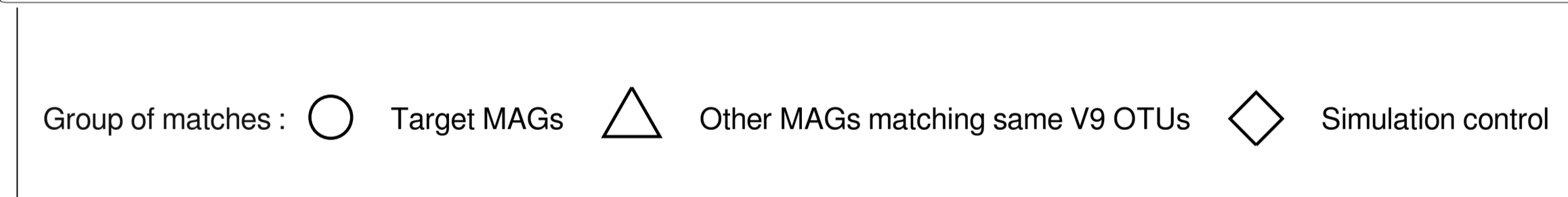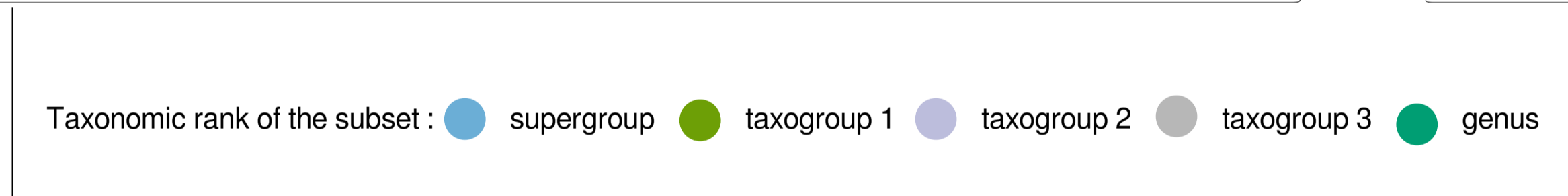

Supplement: S2 File — (ZIP) [file pone.0303697.s002.zip › S9_fig.pdf]

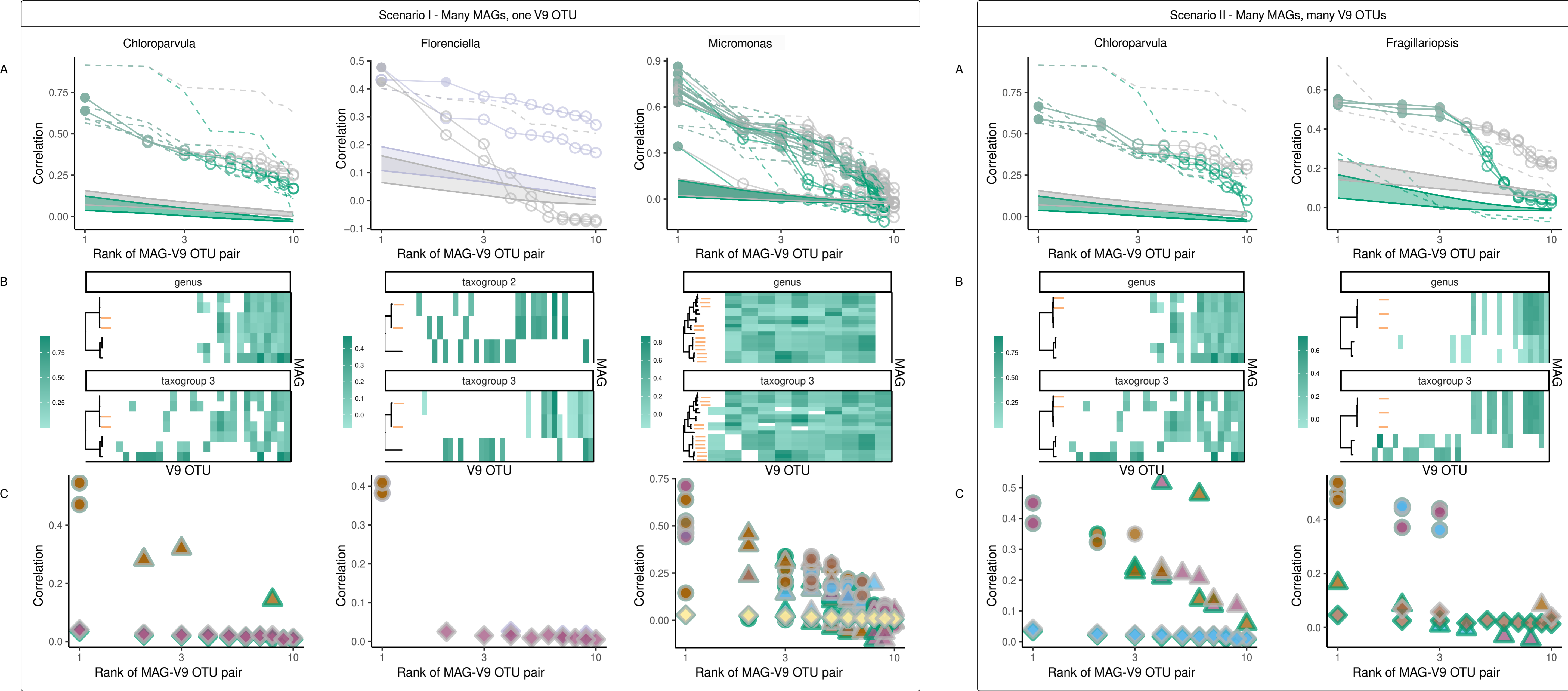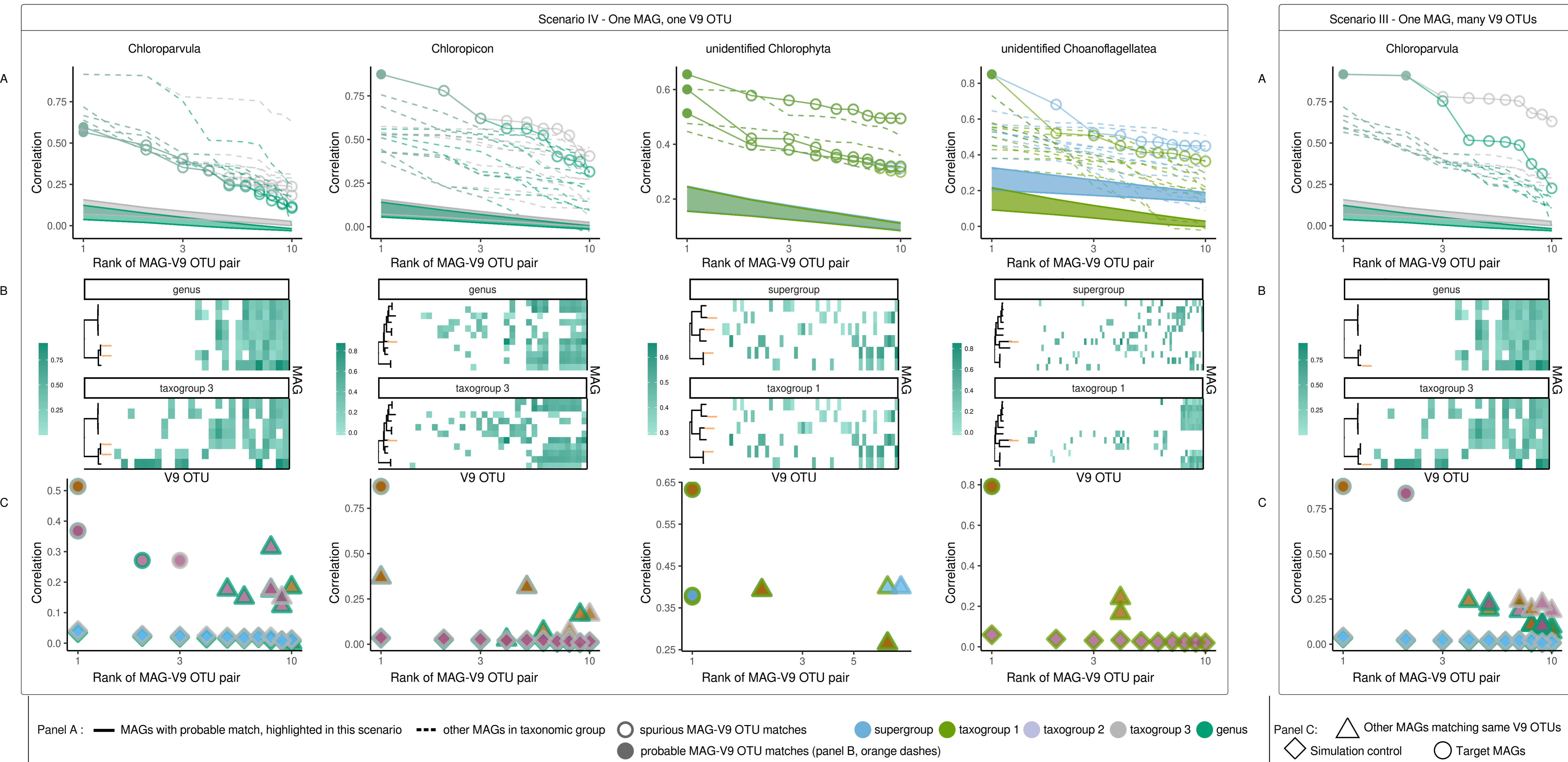

Supplement: S2 File — (ZIP) [file pone.0303697.s002.zip › S10_fig.pdf]
