# Supplementary material for: Diverse patterns of correspondence between protist metabarcodes and protist metagenome-assembled genomes
Source: PLoS One. 2024 Jun 6;19(6):e0303697. doi: 10.1371/journal.pone.0303697 (PMC11156365; doi:10.1371/journal.pone.0303697)
Supplement: Attachment — Submitted filename: barcode_mag_correlation_response_reviewers_revision_1.pdf [file pone.0303697.s003.pdf]

PONE-D-23-39763
Diverse patterns of correspondence between protist metabarcodes and protist metagenome-assembled genomes

Response to Reviewers

We note that all line numbers below refer to the revised manuscript document with tracked changes. This document contains LaTeX code in Microsoft Word format, which we believe is the easiest way to display the differences between the originally submitted version and this revision (despite the fact that it contains some elements of LaTeX code).

Reviewer #1: The work entitled "Diverse patterns of correspondence between protist metabarcodes and protist metagenome-assembled genomes" by Daryna Zavadska and co-workers addresses the question of the consistency of large scale genomic studies, based on concurrent approaches, ie, either from marker gene metabarcoding (processed via the recontruction of OTUs or via ASVs) and whole genome analysis through metagenomics (including single MAG reconstructions). The authors question how these concurrent approaches compare, and if they actually lead to similar results. The comparison is made somehow difficult when some of the markers used are not in *MAG reconstructions, but using reasonable hypotheses, well selected methods, absolutely well mastered statistical analyses and real data, authors ended up with four scenarios.
Scenario I - one V9 OTU matches more than one MAG; Scenario II - more than one V9 OTU matches more than one MAG; Scenario III - more than one V9 OTU matches one MAG; Scenario IV - one V9 OTU matches one MAG. The scientific community postulating that scenario IV is the most likely, should now consider the possibility of scenarii I to III, that may leads to divergent conclusions when treating a same complex environmental sample.
This study is therefore novel, consistent, well designed, well analyzed, and extremely clearly written. I have no critics and no comment.

> We appreciate the reviewer’s comments and we thank them for their time in reviewing our manuscript.

Reviewer #2: In the present work, Zavadska et al. try to match metagenomic assembled genomes and metabarcoding OTUs from the same TARA Oceans samples based on the correspondence of their relative abundances. They first assess which are the best proportionality/correlation metrics to use and build positive and negative controls for validation. Then, after establishing several selection criteria, they obtain 4 different types of MAG-OTU matches, which they discuss. Among other matches, the authors are able to establish reliable one-to-one correspondences in some cases, which represent valuable results to the community.

I think the study is well conducted and methods used are robust, but both methods and results could be better presented in the text and figures (more on this below).

We thank the reviewer for their comments, which helped us to improve our manuscript. We believe we have addressed their concerns related to the methods and results, as described in our responses to each specific point below.

My main concern is that in this study the V9 region (~ 100bp long) of the 18S was used, although samples for the V4 region (~ 380 bp) are also available for the TARA oceans dataset. I think that using the V4 region, which contains nearly 4 times more phylogenetic signal than V9 region, would give different results (and maybe improve the ones already obtained). Thus, I strongly suggest that the authors conduct the same analyses using the V4 dataset.

We thank the reviewer for this suggestion. We ourselves also considered the possibility of using the V4 dataset when we initially planned our analyses. Although the V4 region is longer than the V9, we selected the V9 region for our analyses for the following 3 reasons:

1. The V4 region is more subject to amplification bias than is the V9 (because the V4 has significantly larger variation in length), which could potentially distort the relative abundance values we use as input.
2. The V4 primers used by *Tara* Oceans are less universal than the V9 primers used. The V4 primers are known to exclude some groups of eukaryotes and may exclude other, as yet unknown, groups.
3. At the time we began our analyses, the V4 data had not yet been published. We did not feel it would be appropriate for us to publish a large-scale analysis of unpublished data.

We understand the reviewer's suggestion that V4 could provide different results or improve the ones already obtained, but we believe that adding V4 is not necessary to support the conclusions we derived from the results in our manuscript, for the following 3 reasons:

1. The purpose of our study was not to perform an exhaustive comparison of MAGs versus all other data sources. It was to develop a framework for pairwise comparisons of compositional datasets and, as an example case, to compare MAGs vs. V9 OTUs. In this vein, we also did not compare to other *Tara* Oceans datasets that could provide relative abundance data: metatranscriptomic datasets (within which ribosomal sequences can be collected), or imaging data.
2. Due to the high computational burden of performing the simulations necessary to produce benchmarks for proportionality and correlation values, we estimate that adding a V4 analysis would take 3-4 months, only considering computational time.
3. Variable regions in the 18S do not necessarily evolve at strictly the same rate, even between closely related species within a single eukaryotic lineage. In a small minority of eukaryotic lineages, we expect a perfect one-to-one V4-V9 correspondence. However, in most cases we expect that there is either one V4 for multiple V9s or multiple V4s for one V9. Furthermore, in the case where one V4 corresponds to one V9, it may still hide many different "species" based on ITS or other more variable markers. As the reviewer alluded, this would change the nature of our analyses from a pairwise statistical

comparison, to a multiple statistical comparison among 3 datasets, and would necessitate a new framework to classify and adjudicate differences between V4 and V9. While we agree that this would be a valuable contribution, it would likely require a significant change to our manuscript, and may not necessarily be useful in the near future, as many projects are already adopting long-read metabarcoding approaches that include both variable regions (e.g., Jamy *et al.*, 2022 https://doi.org/10.1038/s41559-022-01838-4; Gaonkar and Campbell, 2024 https://doi.org/10.1002/ece3.11232).

Therefore, although we agree with the reviewer's suggestion that adding V4 could contribute to further knowledge on the relationship between MAGs and barcoding datasets, we believe that adding V4 analyses would be out of the scope of our current work. We would instead suggest that future authors perform comparisons to V4 (or to other datasets), for which we believe our methodology could serve as a basis.

General comments:

The introduction is quite long and repetitive. A more concise writing and, in particular, avoiding repeating ideas in different paragraphs would help. Introduction and Results have several references to the methods used. Given that methods are central in this manuscript, I do not understand why is there no Methods section in the main text. It could be a good idea to create a Methods section with the main ideas instead of putting everything in the Supplementary Material. This could facilitate the reading and give a clearer structure to the manuscript. Also, it could reduce the length of other sections. For example, some lines from Introduction could go there (e.g., lines 53-55, lines 139-144), as well as some lines from the Results (a great portion of the first section, for example). Following with the structure, the Results section contains discussion parts (the discussion on each of the 4 scenarios, for example, which I think is very well presented), but then Discussion has only 3 small paragraphs. I suggest creating a single Results and Discussion section (but this is just a suggestion). Some parts from the introduction could also be moved and expanded in Discussion.

We thank the reviewer for these suggestions. To facilitate reading following the reviewer's advice, we (1) created a new Results and Discussion section, (2) moved parts of the Introduction to the new combined Results and Discussion section, (3) created a Methods section and moved parts of the Introduction and of the Results into the new Methods section, and (4) reorganized the Introduction to remove repetitive sections.

Specific comments:

· L160: where is Figure S4.4? It is constantly referenced in the main text and supplementary information but I did not find it. When clicked, it links to Figure S8. References in the text to this figure does not seem to match Figure S4 (and within Figure S4 there are no numbers but letters for each subfigure). For example, in line 368: 'The final system used to subset SMAGs and V9 OTUs by taxonomy is summarized in Figure S4.4'.

We thank the reviewer for pointing out this error. This was a bug in the LaTeX file that we used to produce the submitted version of the manuscript. We corrected the error in the resubmitted manuscript.

· L177: it can be misleading to reference Figure S6 (and Figure S4.4) when Figures S2-S5 are not referenced yet (they first appear in line 181). Change Figure numbering or text order.

We corrected these errors in figure numbering.

· L180: given that the agreement between rho and Spearman's correlation was used to select candidate matches, these agreeing values need to be in Figure 2 instead of rho proportionality ones. If not, it can be misleading. Also, see specific section below for Figure 2.

We tried the reviewer's suggestion to include Spearman's correlation along with rho proportionality values in Figure 2, but we could not find a way to present all of the data in a clear and readable manner. Instead, we placed all of the Spearman's correlation data in the supplementary figures. We also note that all data necessary to select candidate matches (including proportionality and correlation data) is available in a table on the GitHub accompanying our manuscript, here: https://github.com/beaplab/Protist_barcode_MAG_correspondence/blob/main/data/downstream_outputs/Candidate_pairs-for_R_script_scatterplot_corrected_Pairs_automated.csv

Please see below for responses to other specific suggestions on Figure 2.

· Figure 3: this is a really good summary of the results.

Thank you for this comment, we are glad that the figure provides a useful summary of the overall results.

· Discussion: I miss some discussion on how the sequencing depth could affect the results presented. Are iqlr transformations enough to overcome the limitations of compositional data? Another point worth discussing is that the method used is very dependent on good taxonomic assignments. Finally, I would try to stress the results, as they are relevant to the community. Matching OTUs to MAGs seems to be difficult in most cases, but the authors were able to find a good one-to-one correspondence for some of them. This is really valuable, and I would try to emphasize it in the text.

Thank you for the suggestions. We did not test the effects of sequencing depth in our study, since we used data from a previous paper that directly tested the effects of sequencing depth on the recovery of community diversity in the form of OTUs (Figure 1A and associated supplementary figures from DOI: 10.1126/science.1261605). We added a sentence to the Discussion (lines 852-855) to address the reviewer's suggestion: "A lack of sequencing depth could also result in an underestimate of the sequence diversity present in

a sample, although this is routinely tested in metabarcoding studies and is generally not a significant effect”

Regarding whether iqlr transformations are sufficient to overcome the limitations of compositional data: we agree that although iqlr transformations are theoretically intended to overcome these limitations, it is useful to know whether the approach is valid in practice. Following iqlr transformation, we produced results from both shuffling of actual data and using simulated datasets that showed that we were able to distinguish signal from noise (in which the controls represented a noise signal). Nonetheless, the signal that we were able to detect may be due to an actual biological pattern or may be the results of multiple biological biases. Our results matching V9 OTUs with SAGs (as positive controls) suggest that the biological patterns can indeed be detected for some taxonomic groups, but without a larger number of positive control SAGs, we were not able to test this exhaustively. For other taxonomic groups, we were not able to match V9 OTUs with positive control SAGs, as their proportionality or correlation values were below those of artificially generated noise; in these cases, either the underlying biological signal was not strong enough for our method (including the iqlr transformation) to detect it, or the number of stations in which the SAG was present was too low (as few as only 5 stations), or some combination of the two possibilities (see Supplementary Figure 8).

We completely agree with the reviewer’s point that good taxonomic assignments are critical for our method to work properly. To emphasize this, we added a sentence to the Discussion, lines 864-865: “This highlights the importance of accurate taxonomic assignments to produce optimal results within our approach.”

We also appreciate the reviewer’s point that our approach was able to identify potential one-to-one matches between V9 OTUs and MAGs, and that could be valuable to the community. The philosophy guiding how we prepared our manuscript was to present the results, with appropriate caveats, and to allow the reader to draw their own conclusions based on these results. Therefore, we tried to refrain from overemphasizing the potential V9 OTU and MAG matches that we found in the text, as we believe they represent initial hypotheses for potential relationships. We would not want to lead the reader to believe that we presented strong, direct evidence for any individual match, nor that the overall approach is generally applicable to all taxonomic groups (since we hope to encourage researchers working on specific taxonomic groups to investigate the results of our approach within that particular group).

· L311: reference?

Although the idea that either V9 OTUs or MAGs are more appropriate representations of underlying biological entities is implied in various articles in the literature, we were unable to find any references that explicitly assert the superiority of one or the other approach (instead, it is generally implicit, in our interpretation). To reduce the possibility of confusion, we removed this sentence from our article.

· L350: I really appreciate that the authors are sharing all data and code. This should be the way to work in every manuscript, but unfortunately it is not.

We completely agree.

· L380: why this filtering?

We removed V9 OTUs with total abundance less than 100 as they were not likely to contribute any signal to our measurements of proportionality or correlation. This is reflected in the figure below, where total abundance for reads assigned to a given OTU is on the horizontal axis and that OTU's maximum relative abundance in a single sample is on the vertical axis. As can be observed, OTUs with total abundance less than 100 essentially never contribute substantially to relative abundance in any sample:

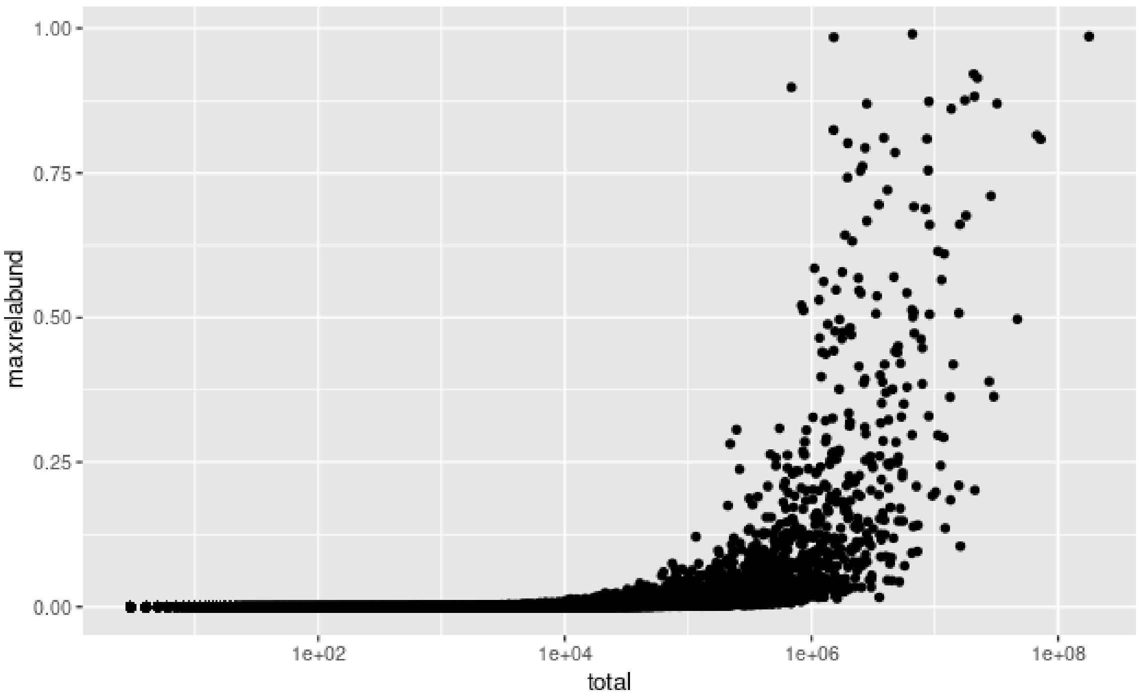

To address the reviewer's comment, we added the following to the supplementary information (lines 1339-1342): "we removed V9 OTUs whose sum of number of reads across all samples was ≤ 100 from subsequent calculations, as these OTUs did not contribute substantially to relative abundance in any sample (data not shown)." We note that we corrected a typo in this sentence, in which the threshold was erroneously written to be ≥ 100, not ≤ 100. We thank the reviewer for pointing out this error.

· L424: which values/thresholds were used to create these categories?

These categories were assigned heuristically in a human-curated manner, in order to create consistent groupings for interpretation. Although we initially attempted to define categories in an automated way, we could not, for three reasons:

1. Empirical scores vary greatly depending on the number of MAGs and V9s assigned to a particular taxogroup, and on the precision of taxonomic identification of entities within the taxogroup.
2. Uniqueness scores from a given taxogroup do not always reflect similar underlying biological realities. If the same V9 OTU is considered a high-consistency match in one taxogroup, and low-consistency match in another, this makes a true match highly probable for the former case, and it is likely an artifactual match in the latter. In contrast, if the same V9 matches equally well to MAGs from two different taxogroups with the highest consistency, this makes both matches low confidence, although consistency scores themselves would point to high confidence for both. The uniqueness score in both scenarios described will be the same, meaning that use of the score itself cannot be converted easily to a simple threshold.
3. For some cases, in particular for high-probability matches, relative abundance scatterplots were manually checked to additionally verify that the match was not an artifact.

To clarify this point, we modified the text of the supplementary information as follows (lines 1407-1416): “Depending on the consistency between taxonomic subsets and metrics, and on the uniqueness of V9 OTU match within/outside of a SMAG taxonomic genre, different categories reflecting the quality of the SMAG-V9 OTU match were assigned heuristically to each pair: "Less Probable" (very low confidence) < "Probable" (low confidence) < "More Probable" (medium confidence) < "Yes" (high confidence). We note that the final list of potentially matching pairs was assessed manually and is not meant to be exhaustive. The complete list is available on the GitHub associated with this manuscript.”

· Figure 2:

I suggest rethinking and redoing Figure 2. This figure tries to summarize so much information in a single page that it is really hard to understand. At least, it was really difficult for me to follow everything that is presented. Given that these are case examples (and not all results), it could be better to just show one representative case per scenario and save the rest for supplementary. With this, plots could be a little bit bigger and overlapping data/lines/points could be separated into different plots.

We agree with the reviewer that Figure 2 was in need of simplification. To this end, we clarified each panel, and we removed panel (c), in order to provide more space for the other three panels. In addition, we changed elements in each panel to make the information easier to view and interpret, as described in detail below. We tested the possibility of including only one representative case for each scenario, but we found that multiple examples would be more useful, to illustrate the differences that can be detected even within a given scenario.

In a) all top 10 matches are shown equally and only the dots are highlighting the MAGs selected in each scenario. This is hard to interpret, as dots have too much overlap to see a clear picture. For example, the Chloroparvula plot is repeated 4 times and only the dots change (and there is a lot of overlap, so these are hard to see separately). I would make the separation between matches considered and not considered in each scenario much clearer. Having the 2 taxonomic levels mixed in the same plot is also not helpful, I would separate them.

We thank the reviewer for these suggestions. We modified panel (a) such that the individual MAGs represented and the MAGs highlighted in other panels should now be easier to distinguish. Specifically, following the reviewer's suggestion, to make the separation between matches considered and not considered in each scenario more clear, we modified both the lines representing MAGs and the points representing MAG-V9 OTU pairs. Solid lines now represent MAGs highlighted in a given scenario, and MAGs not represented are shown in dashed lines. Points representing MAG-OTU pairs highlighted in other panels are depicted with filled circles, and non-highlighted pairs are shown in empty circles. We also adjusted the transparency of both points and lines to make overlapping items easier to view. In the case of *Chloroparvula*, we believe that our modifications should clarify that different MAGs classified within the same group are found in different scenarios, with the MAGs from alternative scenarios found in the same figure for ease of comparison.

We also tested the possibility of including on a single taxonomic level in each plot, rather than 2 taxonomic levels, but we found that including both levels was useful in order to compare between the taxonomic levels, as it was difficult to compare between levels when the plots were side-by-side rather than superimposed.

In b), tiles are so little in some cases that are impossible to read. Also, is there some information repeated between a) and b)? This is not clear to me. Why does Florenciella have different taxonomic levels than the rest (Scenario I, section b)? This is hard to find in the text.

To address the reviewer's suggestions for the modifications of this panel, we changed the orange highlighting for a given MAG by removing the border around individual rows in the shaded matrix, and indicating MAGs highlighted from panel (a) as thick orange horizontal lines next to the trees on the left side of the shaded matrix. This allowed us to enlarge the individual tiles within the matrix. We also changed the gradient of the matrix from two colors to a single color, in order to make it easier to perceive. We believe these changes should allow the reader to understand which information is repeated between panels (a) and (b), and why it is repeated.

In order to clarify how different groups may have different taxonomic levels represented, we added the following sentence to the figure caption for panel (a) (lines 680-682): "Different groups have different taxonomic levels represented, depending on the resolution of the taxonomic assignments of the V9 OTUs in the group (see Table S1)."

In the specific case of *Florenciella*, although it is a genus, the taxonomic resolution of its V9 OTUs was above the genus level (see Table S1).

In c), fill colors overlap with colors of taxonomic ranks. Also, the outline of the points is hardly visible. And in some cases, the overlap is so high that the plot cannot be interpreted. Could the dots be connected to make it clearer? This needs improvement.

We appreciate the reviewer's suggestions to improve panel (c). We tried each of the suggestions. We corrected the colors to remove overlaps of fill colors with those of taxonomic ranks. We increased the size of the outline of the points (and the sizes of the points themselves). To reduce information loss due to overlaps, we increased the transparency of points. We tried the suggestion to connect points to increase clarity, but we found that this introduced potential confusion into the plot, as the lines could overlap, and since points of a single group can occur in both the horizontal and vertical axis, resulting in both horizontal and vertical lines.

We note that panel (c) has now been moved to the supplement, in order to make the other panels larger and easier to view.

In d), what do the colors represent?

We agree that the color scheme was in need of clarification. Each color shows a separate MAG or V9 OTU. Although the colors are not strictly necessary, as they do not encode additional information, we found it more intuitive to use colors in order to highlight that each subplot represents a different MAG-V9 OTU pair with the use of different colors.

In order to clarify the meaning of the colors in this panel (C, previously panel D in the initial submission), we modified the caption of the figure (line 710), as follows: "Different colours represent different MAGs and V9 OTUs."
